# Influence of Topic Familiarity and Prompt Specificity on Citation Fabrication in Mental Health Research Using Large Language Models: Experimental Study

**DOI:** 10.2196/80371

**Published:** 2025-11-12

**Authors:** Jake Linardon, Hannah K Jarman, Zoe McClure, Cleo Anderson, Claudia Liu, Mariel Messer

**Affiliations:** 1 School of Psychology Faculty of Health Deakin University Geelong, Victoria Australia

**Keywords:** large language models, artificial intelligence, AI, mental health, psychiatry, citations, academic research

## Abstract

**Background:**

Mental health researchers are increasingly using large language models (LLMs) to improve efficiency, yet these tools can generate fabricated but plausible-sounding content (*hallucinations*). A notable form of hallucination involves fabricated bibliographic citations that cannot be traced to real publications. Although previous studies have explored citation fabrication across disciplines, it remains unclear whether citation accuracy in LLM output systematically varies across topics within the same field that differ in public visibility, scientific maturity, and specialization.

**Objective:**

This study aims to examine the frequency and nature of citation fabrication and bibliographic errors in GPT-4o (Omni) outputs when generating literature reviews on mental health topics that varied in public familiarity and scientific maturity. We also tested whether prompt specificity (general vs specialized) influenced fabrication or accuracy rates.

**Methods:**

In June 2025, GPT-4o was prompted to generate 6 literature reviews (~2000 words; ≥20 citations) on 3 disorders representing different levels of public awareness and research coverage: major depressive disorder (high), binge eating disorder (moderate), and body dysmorphic disorder (low). Each disorder was reviewed at 2 levels of specificity: a general overview (symptoms, impacts, and treatments) and a specialized review (evidence for digital interventions). All citations were extracted (N=176) and systematically verified using Google Scholar, Scopus, PubMed, WorldCat, and publisher databases. Citations were classified as fabricated (no identifiable source), real with errors, or fully accurate. Fabrication and accuracy rates were compared by disorder and review type by using chi-square tests.

**Results:**

Across the 6 reviews, GPT-4o generated 176 citations; 35 (19.9%) were fabricated. Among the 141 real citations, 64 (45.4%) contained errors, most frequently incorrect or invalid digital object identifiers. Fabrication rates differed significantly by disorder (*χ*^2^_2_=13.7; *P*=.001), with higher rates for binge eating disorder (17/60, 28%) and body dysmorphic disorder (14/48, 29%) than for major depressive disorder (4/68, 6%). While fabrication did not differ overall by review type, stratified analyses showed higher fabrication for specialized versus general reviews of binge eating disorder (11/24, 46% vs 6/36, 17%; *P*=.01). Accuracy rates also varied by disorder (*χ*^2^_2_=11.6; *P*=.003), being lowest for body dysmorphic disorder (20/34, 59%) and highest for major depressive disorder (41/64, 64%). Accuracy rates differed by review type within some disorders, including higher accuracy for general reviews of major depressive disorder (26/34, 77% vs 15/30, 50%; *P*=.03).

**Conclusions:**

Citation fabrication and bibliographic errors remain common in GPT-4o outputs, with nearly two-thirds of citations being fabricated or inaccurate. Reliability systematically varied by disorder familiarity and prompt specificity, with greater risks in less visible or specialized mental health topics. These findings highlight the need for careful prompt design, rigorous human verification of all model-generated references, and stronger journal and institutional safeguards to protect research integrity as LLMs are integrated into academic practice.

## Introduction

Researchers today face growing pressure to maintain high levels of productivity to remain competitive for funding, tenure-track positions, academic promotions, and international recognition in their fields [1]. This intensifying demand for outputs—which typically spans research, teaching, supervision, and service—has led to a need for innovative solutions that can streamline administrative and research-related workflows. As the academic workload becomes increasingly complex and resource constrained, tools that can support efficiency without compromising quality are gaining significant attention.

Large language models (LLMs) are a class of tools gaining traction in academic settings to support researchers. LLMs are advanced artificial intelligence systems trained on vast amounts of textual data to generate coherent and contextually relevant natural language responses [2]. Among the many LLMs available, ChatGPT (OpenAI) has emerged as the most widely used, both in the general population [3] and among researchers [4,5]. A recent study showed that nearly 70% of mental health scientists reported using ChatGPT to assist with research-related tasks, including writing and drafting, coding, and administrative support [4]. However, while most LLM adopters reported that these tools enhanced research efficiency and improved the quality of their work, many also expressed concerns about inaccuracies, misleading content, and biases in the responses generated by these models [4]. These concerns are not unfounded, as one of the most well-documented limitations of LLMs is their tendency to “hallucinate,” which occurs when the model generates false, fabricated, or unverifiable information that appears coherent and credible [6].

One type of hallucination generated by LLMs that has received increasing attention among researchers is fabricated bibliographic citations that cannot be traced to existing scholarly publications. This is a critical issue reported in LLM output because citations serve as the foundation of scholarly communication—they guide readers to source evidence; build cumulative knowledge; and inform research, policy, and clinical practice. Fabricated references can mislead readers, distort scientific understanding, and compromise the integrity of academic work.

Recent work has sought to quantify the extent of these hallucinations, typically by analyzing the citations produced when LLMs are prompted to generate academic literature reviews on specific topics. Walters and Wilder [7] prompted GPT-3.5 and GPT-4 to generate short literature reviews on 42 multidisciplinary topics and found that 55% and 18% of the citations, respectively, were fabricated, and 43% (GPT-3.5) and 24% (GPT-4) of the real citations contained substantive errors. Mugaanyi et al [8] compared citation fabrication rates in GPT-3.5–generated literature reviews across disciplines by analyzing 5 reviews each in the natural sciences and humanities. They found that while there were no differences in fabricated citation rates between the 2 disciplines (27.3% vs 23.4%), the rate of digital object identifier (DOI) hallucinations among the generated citations was higher for humanities (89.4%) than for natural sciences (61.8%) [8]. In another study, McGowan et al [9] used Google’s Bard (now Gemini) to assess citation accuracy when prompted to generate references for research topics that were broad (ie, evidence for hallucinations in schizophrenia) versus specific (ie, research using natural language processing to assess suicidal behavior) in nature. When prompted on the broader topic, none of the 10 citations generated by Bard were accurate, with several linking to entirely unrelated papers. In contrast, when prompted on the specific topic, all 5 citations contained misattributed authorship but linked to real papers with matching titles [9]. These findings collectively demonstrate that both the disciplinary domain and the specificity of the prompt can influence the likelihood and nature of citation hallucinations, highlighting the need for systematic evaluations of LLM citation reliability across varied research contexts.

In this study, we provide a more nuanced examination of citation fabrication in the most recent version of GPT (GPT-4o; Omni) outputs by systematically varying the level of public awareness and depth of existing scientific literature on the topic within the domain of mental health. In particular, we prompted GPT-4o to generate academic literature reviews for 3 distinct mental disorders that differ in public visibility and research maturity. We selected major depressive disorder, binge eating disorder, and body dysmorphic disorder because they reflect varying levels of clinical familiarity and scientific development within the mental health field. Major depressive disorder is widely studied and publicly recognized, with a substantial volume of research and clinical trial activity. Binge eating disorder occupies an intermediate position in terms of research output and public awareness, whereas body dysmorphic disorder remains comparatively understudied and less widely known [10]. These classifications are supported by differences in publication volume, clinical trial prevalence, and general population familiarity across the 3 disorders. We also varied the level of prompt specificity, asking GPT-4o to generate either a general overview of each disorder (including its symptoms, societal impacts, and treatment approaches) or a specialized review focused on the narrower topic (the evidence base for digitally delivered interventions).

Given that LLMs are trained on large-scale text corpora comprising publicly available and licensed content [2], it is plausible that topics with lower online visibility, limited research coverage, or highly specialized scope are more prone to citation-related hallucinations. This may be because such topics are underrepresented in the training data and often lack readily accessible, high-quality academic content in the public domain, thereby reducing the model’s ability to retrieve accurate and verifiable sources. The novelty of this study is that it goes beyond previous cross-disciplinary examinations of citation hallucinations to provide the first within-domain test of how topic familiarity and prompt specificity shape LLM reliability. By manipulating both the disorder under review and the granularity of the task, we were able to generate new insights into when and why citation hallucinations are more likely to occur in scientific contexts. Therefore, we aimed to examine whether citation fabrication and bibliographic accuracy in GPT-4o outputs systematically vary by disorder type and review specificity.

## Methods

### Design

We used GPT-4o to generate brief literature reviews on 6 different topics and then aggregated data on the citations generated in the papers. We then searched online databases and websites to examine the frequency of (1) fabricated citations, (2) errors in the citations of nonfabricated sources, and (3) completely accurate citations among the nonfabricated sources. Multimedia Appendix 1 presents the prompts given for the 6 review topics, the text generated by GPT-4o, and the full citations provided in the output.

### Review Topics and Prompts

GPT-4o was tasked (June 2025) with generating a short literature review on 6 distinct topics. For each prompt, a new session was initiated, and chat history was cleared to eliminate any influence from previous interactions. The topics spanned 3 different mental disorders that varied in public knowledge, volume of scientific research, and the level of specificity of the research questions provided to GPT-4o. For the general overview, GPT-4o was asked to write about the causes, societal and economic impacts, and available treatment approaches for 1 of the 3 disorders (major depressive disorder vs binge eating disorder vs body dysmorphic disorder). For the specialized reviews, GPT-4o was asked to synthesize the evidence base for digital interventions and discuss the factors that may influence their effectiveness. We selected digital interventions as the specialized topic because all 3 disorders have a growing yet uneven body of research in this area, making it a suitable test case for examining GPT-4o’s ability to retrieve and summarize nuanced evidence across conditions that differ in research maturity. For example, more than 100 clinical trials have evaluated digital interventions for depression [11] compared to approximately a dozen for binge eating disorder [12] and an even smaller number for body dysmorphic disorder [13].

Prompt development was guided by 2 principles: replicability and ecological validity. First, we closely mirrored the style of prompts used in previous studies of LLM-generated literature reviews in other disciplines [7]. Second, we sought to design prompts that reflected how researchers might realistically request literature reviews from an LLM in practice. Draft prompts were discussed extensively among all authors and revised to ensure consistency, neutrality of tone, and appropriateness for the study aims. Importantly, we did not test these prompts within GPT-4o during development to avoid introducing previous knowledge or contamination from earlier runs. While advanced prompt engineering strategies (eg, chain of thought, few-shot examples, and retrieval-augmented prompts) were considered, these were not implemented, as our study sought to assess citation reliability under straightforward prompting conditions that approximate typical researcher use.

We asked GPT-4o to simulate an academic researcher and write a literature review of approximately 2000 words, using at least 20 citations from peer-reviewed academic sources, preferably within the last 10 years. Two major prompts were used, 1 generic and 1 specialized, and were held constant across reviews, with only the target disorder substituted in the text. Prompts, outputs, and citations generated by GPT-4o are presented in Multimedia Appendix 1.

### Data Collection and Analysis

For the 6 literature reviews generated by GPT-4o, we recorded the number of words generated, number of citations provided, frequency of sources provided (eg, journal articles and books), and the full bibliographic information. For each citation, we first searched Google Scholar to see whether it was real or fabricated. If no matching source was found, we then searched using the full author list to identify any resembling publications. If this was also unsuccessful, we searched using the full title of the citation. If the citation still could not be verified, we repeated the process across other databases, including Google, Scopus, PubMed, and WorldCat. As a final step in verifying potentially fabricated works, we manually reviewed the relevant journal volume and issue and used the publisher’s website search function to confirm that the cited work did not exist.

We considered a citation to be genuine (not fabricated) if we could identify a real publication that closely matched both the title and the authors. In other words, inaccuracies in the citation were permitted if the cited work could be reliably traced to an actual source. For example, a GPT-4o citation that listed the correct authors and title but included an incorrect volume number, page range, or DOI was still considered a real (nonfabricated) source. For these nonfabricated sources, we then compared the GPT-4o citation to the actual citation and recorded whether there were any discrepancies and errors in the author list, year, title, journal, volume, issue, page range, and DOI. This did not include formatting errors (eg, italicized journal names). We then recorded the number of GPT-4o real (nonfabricated) citations that contained no errors in any of the bibliographic details.

Fabrication and accuracy rates were calculated as proportions and expressed as percentages. These rates were presented descriptively overall and separately by target disorder (major depressive disorder vs binge eating disorder vs body dysmorphic disorder) and review type (general vs specialized).

To examine whether fabrication and accuracy rates varied by disorder and review type, we conducted a series of chi-square tests. Specifically, we tested for main effects by evaluating the association between review type and fabrication and accuracy rates and between disorder and fabrication and accuracy rates across all reviews.

Although interaction effects were not formally modeled because of low expected cell counts in some conditions, we conducted stratified chi-square tests to explore potential group differences within each level of the independent variables, that is, we examined whether review type (general vs specialized) was associated with fabrication or accuracy rates within each of the 3 diagnostic subgroups and whether diagnostic subgroup was associated with fabrication or accuracy rates within each review type.

All chi-square tests were 2 tailed, and pairwise comparisons were conducted where omnibus tests were significant. Significance was set at *P*<.05.

### Ethical Considerations

This study was exempt from ethical review as no human participants were involved.

## Results

### Overview

The number of words generated by GPT-4o across the 6 literature reviews ranged from 1096 (specialized review for binge eating disorder) to 1326 (general review for binge eating disorder). The total number of references provided by GPT-4o was 176, which ranged from 17 (specialized review for body dysmorphic disorder) to 36 (generic review for binge eating disorder) across the 6 conditions (Table 1). Most citations provided by GPT-4o were journal articles, with books and book chapters comprising the rest. Multimedia Appendix 2 provides the citations provided by GPT-4o, along with the correct citation identified by the author team.

**Table 1:** Prevalence of fabricated and accurate citations generated by ChatGPT-4o overall and by literature review and diagnostic subtype.

Variable		Major depressive disorder, n/N (%)			Binge eating disorder, n/N (%)			Body dysmorphic disorder, n/N (%)			Combined references, n/N (%)		
		General (n=35 references; 1302 words)	Specialized (n=33 references; 1146 words)	General (n=36 references; 1326 words)		Specialized (n=24 references; 1096 words)	General (n=31 references; 1225 words)		Specialized (17 references; 1239 words)	General (n=102)		Specialized (n=74)	Total (N=176)
Fabricated references		1/35 (2.9)	3/33 (9.1)	6/36 (16.7)		11/24 (45.8)	10/31 (32.3)		4/17 (23.5)	17/102 (16.7)		18/74 (24.3)	35/176 (19.9)
Accurate references^a^		26/34 (76.5)	15/30 (50)	18/30 (60)		8/13 (61.5)	3/21 (14.3)		7/13 (53.8)	47/85 (55.3)		30/56 (53.3)	77/141 (54.6)
**Error type^b^**													
	Author list	3/34 (8.8)	5/30 (16.7)	3/30 (10)		0 (0)	5/21 (23.8)		5/13 (38.5)	11/85 (12.9)		10/56 (17.9)	21/141 (14.9)
	Year	5/34 (14.7)	7/30 (23.3)	9/30 (30)		3/13 (23.1)	5/21 (23.8)		4/13 (30.8)	19/85 (22.4)		14/56 (25)	33/141 (23.4)
	Title	0 (0)	4/30 (13.3)	5/30 (16.7)		1/13 (7.7)	9/21 (42.9)		3/13 (23.1)	14/85 (16.5)		8/56 (14.3)	22/141 (15.6)
	Journal	0 (0)	7/30 (23.3)	3/28 (10.7)		0/0 (0)	8/18 (44.4)		2/11 (18.2)	11/79 (13.9)		9 /53 (17)	20/132 (15.2)
	Volume	4/33 (12.1)	10 /30 (33.3)	7/27 (25.9)		3/12 (25)	12/18 (66.7)		3/10 (30)	23/78 (29.2)		16 (30.8)	39/130 (30)
	Issue	4/32 (12.5)	9/30 (30)	7/27 (25.9)		3/12 (25)	10/18 (55.6)		3/10 (30)	21/77 (27.3)		15/52 (28.8)	36/129 (27.9)
	Page	5/33 (15.2)	9/30 (30)	7/27 (25.9)		3/12 (25)	11/18 (61.1)		4/11 (36.4)	23/78 (29.5)		16/52 (30.2)	39/131 (29.8)
	DOI^c^	6/33 (18.2)	11/30 (37.7)	9/28 (32.1)		5/13 (38.5)	14/19 (73.7)		6/12 (50)	29/80 (36.3)		22/55 (40)	51/135 (37.8)
Journal article		34/35 (97.1)	33/33 (100)	33/ 36 (91.7)		23/24 (95.8)	27/31 (87.1)		15/17 (88.2)	94/102 (92.2)		71/74 (95.6)	165/176 (93.8)

^a^Percentage derived for accurate references does not factor in hallucinated references in the denominator. Denominator varies based on whether information was provided in the citation generated by ChatGPT (eg, missing volume number for one citation was not included in the denominator for accuracy rate for overall volume numbers).

^b^Error type was only relevant for actual references. Sources that did not include specific information to form an American Psychological Association reference (eg, volume, issue, journal, and page number for books) were counted as missing and did not contribute to the denominator.

^c^DOI: digital object identifier.

### Fabrication Rates

Of the 176 citations provided by GPT-4o, 35 (19.9%) were fabricated. When GPT-4o provided a DOI for a fabricated citation, 64% (21/33) of the DOIs were valid but linked to irrelevant and unrelated journal articles, whereas 36% (12/33) were completely invalid or nonfunctional.

The number of fabricated sources overall did not significantly differ between general (17/102, 16.7%) and specialized (18/74, 24%) literature reviews (*χ*^2^_1_=1.6; *P*=.21). However, when conducting stratified analyses by diagnostic subgroup, a significant association between literature review type and fabrication rate emerged only for binge eating disorder: fabrication rate was significantly higher for specialized (11/24, 46%) compared to generic (6/36, 17%) reviews on binge eating disorder produced by GPT-4o (*P*=.01).

When comparing the number of fabricated sources overall between the 3 diagnostic subtypes, a significant overall difference was observed (major depressive disorder: 4/68, 6%; binge eating disorder: 17/60, 28%; body dysmorphic disorder: 14/48, 29%; *χ*^2^_2_ =13.7; *P*=.001). Pairwise comparisons show that the fabrication rate was significantly higher for binge eating disorder compared to major depressive disorder (*P*=.001) and for body dysmorphic disorder compared to major depressive disorder (*P*=.001), but no difference emerged when comparing binge eating disorder to body dysmorphic disorder (*P*=.92). When conducting stratified analyses by review type, significant differences in fabrication rates were found between the 3 diagnostic subtypes for both general (*P*=.006) and specialized (*P*=.006) reviews. Within generic reviews, fabrication rates were significantly lower for major depressive disorder than for body dysmorphic disorder (*P*=.001). Within specialized reviews, fabrication rates were significantly higher for binge eating disorder than for major depressive disorder (*P*<.001).

### Accuracy Rates

Only 77 (54.6%) of the 141 real (nonfabricated) citations provided by GPT-4o were accurate and contained no errors. The number of accurate citations overall did not significantly differ between general (47/85, 55%) and specialized (30/56, 54%) literature reviews (*χ*^2^_1_ =0.4; *P*=.84). However, when conducting stratified analyses by diagnostic subtype, differences in accuracy rates between review types did emerge. Within major depressive disorder, accuracy was significantly higher for generic (26/34, 77%) compared to specialized (15/30, 50%) reviews (*P*=.03), whereas within body dysmorphic disorder, accuracy was significantly higher for specialized (7/13, 54%) compared to generic (3/21, 14%) reviews (*P*=.01).

When comparing accuracy rates overall between the 3 diagnostic subtypes, a significant overall difference was observed (major depressive disorder: 41/64, 64%; binge eating disorder: 26/43, 60%; body dysmorphic disorder: 10/34, 29%; *χ*^2^_2_ =11.6; *P*=.003). Pairwise comparisons show rates of accurate citations were significantly higher for major depressive disorder compared to body dysmorphic disorder (*P*=.001) and for binge eating disorder compared to body dysmorphic disorder (*P*=.007) but not when comparing major depressive disorder to binge eating disorder (*P*=.71). When conducting stratified analyses by review type, significant overall differences in accuracy rates between diagnostic subtypes emerged only for generic reviews (*P*<.001). Specifically, accuracy rates within generic reviews were significantly lower for body dysmorphic disorder compared to both major depressive disorder (*P*<.001) and binge eating disorder (*P*=.001).

### Citation Errors

Table 1 also provides the percentage of specific errors observed across real (nonfabricated) citations overall and by condition. Combined, the highest error rate was observed for DOIs for journal articles (51/141, 36.2%), and the lowest error rate was observed for the author list (21/141, 14.9%). Similar trends were observed when inspecting error rates across the 6 study conditions (Table 1).

## Discussion

### Principal Findings

This study examined whether citation fabrication and bibliographic accuracy in GPT-4o output systematically vary across topic areas of different public visibility, scientific development, and specialization. We prompted GPT-4o to generate literature reviews on 3 psychiatric conditions (ie, major depressive disorder, binge eating disorder, and body dysmorphic disorder) that vary in public knowledge and research maturity. The literature reviews generated by GPT-4o also varied in scope, comprising both general overviews of each disorder (including symptoms, impacts, and treatment approaches) and highly specific reviews focused on the efficacy and moderators of digitally delivered interventions for these disorders. Given that LLMs are trained on large-scale text corpora comprising publicly available content [2], we expected that literature reviews focused on more widely recognized disorders (ie, major depressive disorder) and those addressing general topics would yield lower rates of citation fabrication and bibliographic errors compared to reviews on less familiar disorders or more specialized subfields.

Our expectations were mostly supported. Overall, GPT-4o generated 176 citations from 6 literature reviews. A total of 35 (19.9%) citations were fabricated, and among the 141 nonfabricated citations, 77 (54.6%) contained bibliographic errors, with incorrect DOIs being the most prevalent error type. Fabrication and accuracy rates varied by disorder, with the lowest fabrication rates observed in reviews on major depressive disorder and the lowest citation accuracy rates found in reviews on body dysmorphic disorder. While citation fabrication and accuracy rates did not differ *overall* by review type, stratified analyses revealed important within-disorder differences. First, fabrication rates were higher in specialized than general reviews within binge eating disorder. Second, citation accuracy was higher for general than specialized reviews within major depressive disorder. Third, accuracy was unexpectedly higher in specialized than general reviews within body dysmorphic disorder. Collectively, these findings demonstrate that both the subject area and prompt granularity can influence the likelihood and nature of citation hallucinations and the accuracy generated by LLMs.

### Comparisons With Prior Work

The findings of this study align with previous research across various disciplines showing that citation fabrication and bibliographic inaccuracies are common in LLM-generated outputs [7-9,14]. However, this study extends this work in several important ways. First, while previous research has compared model outputs across distinct disciplines [7,8], which has made it difficult to draw conclusions about relative performance across topics of differing complexity within the same field, we examined how citation reliability varies within the same domain by systematically manipulating public familiarity, scientific visibility, and prompt granularity. This approach enabled a more fine-grained analysis of how topic characteristics influence LLM performance, revealing meaningful variation even within a single area of research. Second, while most earlier studies evaluated older versions of GPT [8,15,16], our use of GPT-4o allowed us to assess whether citation reliability has improved in newer iterations. Despite expectations of enhanced accuracy, we found no clear evidence of reduced hallucination rates, although cross-study comparisons are inherently limited due to variation in topic selection, task framing, and evaluation methods.

A key novelty of this work in comparison to previous literature lies in its design. Rather than contrasting different disciplines or older model versions, we systematically manipulated topic familiarity and review specificity within the same research field. This approach revealed important topic-level differences in citation reliability that would have been obscured in cross-disciplinary comparisons. Thus, this study not only extends existing evidence on LLM hallucinations but also introduces a new framework for evaluating how characteristics of the research question itself influence model performance.

### Limitations

This study has several limitations that should be acknowledged. First, although a key strength of this study was the manipulation of topic complexity and public familiarity within a single domain, the findings may not generalize to other psychiatric disorders or specialized subfields not examined here. Future research should assess whether similar patterns of citation fabrication and bibliographic errors are observed across a broader range of mental health topics and other academic disciplines.

Second, our findings are specific to GPT-4o and may not generalize to outputs generated by other LLMs. We selected GPT-4o because it is the most recent and widely adopted LLM among academic researchers [4], making our results relevant to a significant proportion of users. Nevertheless, further studies should investigate whether different LLMs exhibit comparable patterns or whether model-specific factors influence citation reliability.

Third, we did not use prompt engineering strategies; instead, we used standardized but straightforward prompts to reflect typical researcher use. It is possible that performance could be improved through techniques such as citation verification prompts or structured reference formatting. Future studies should directly test whether such prompt engineering methods reduce hallucination and error rates.

Fourth, we analyzed a single output per prompt and did not assess intraprompt variability across multiple generations. As LLMs such as GPT-4o produce outputs stochastically, it is possible that replication of the same prompt would produce different citation fabrication or accuracy rates. Future work should examine multiple generations per prompt to assess the consistency and reproducibility of results.

### Broader Implications

This research has broader implications for the conduct of scientific research. First, researchers and students should be aware of the risks associated with relying on LLMs for literature generation and ensure that all outputs are subjected to careful human oversight. This includes systematically checking, validating, and verifying the accuracy and authenticity of any citations or claims produced by models. Without such oversight, the field risks the proliferation of fabricated or inaccurate references, which can erode scientific integrity and mislead readers, reviewers, and the public.

Second, journal editors and publishers have a critical role to play in maintaining scholarly standards by ensuring that LLM-generated citations do not make their way into published outputs unchecked. One practical strategy is to use plagiarism detection software that flags whether citations appear in known published sources. If a citation is identified by such software as plagiarized or matching existing content, this can serve as a positive indicator that the reference is real. Conversely, if a citation does not trigger any match, it may signal that the source does not exist and was potentially hallucinated by the LLM, warranting closer inspection and manual verification.

Third, academic institutions and research organizations should develop clear policies and training guidelines around the responsible use of LLMs in scientific writing. As these tools become more deeply integrated into research workflows [5], it is essential to equip users with the knowledge and skills to critically assess model-generated outputs. This includes instruction on how to identify hallucinated citations, validate bibliographic content, and appropriately disclose the use of generative artificial intelligence in scholarly outputs.

### Conclusions

In conclusion, this research offers novel insights into how the characteristics of a topic influence the reliability of citations produced by LLMs. While we found that citation fabrication and error rates are common overall, these inaccuracies were less frequent when prompts addressed topics with greater public awareness, an established evidence base, and a broad scientific consensus. This suggests that the reliability of LLM-generated citations is not fixed but contingent on the informational terrain they are asked to navigate. Importantly, these findings have broader implications for the integration of LLMs into scholarly workflows. They indicate that improving citation accuracy is not solely a technical challenge but also a matter of strategic prompt design and topic selection. Researchers can leverage this insight using LLMs preferentially for well-established domains while exercising caution and implementing verification protocols when working in specialized areas where training data may be sparse or inconsistent. More broadly, our results highlight the need for institutional guidance and training that explicitly addresses the contextual factors influencing LLM reliability rather than treating citation errors as random or inevitable. By embedding such practices, the academic community can harness LLMs’ efficiencies while safeguarding the integrity of scholarly work.

## Appendix

Multimedia Appendix 1 ChatGPT prompts and outputs across the 3 disorders and prompt specificity.

Multimedia Appendix 2 Comparison of GPT citation outputs with actual citations.

## Abbreviations

DOI: digital object identifier
LLM: large language model

## Data Availability

The datasets generated or analyzed during this study are available from the corresponding author on reasonable request.
