# Supplementary material for: Influence of Topic Familiarity and Prompt Specificity on Citation Fabrication in Mental Health Research Using Large Language Models: Experimental Study
Source: JMIR Ment Health. 2025 Nov 12;12:e80371. doi: 10.2196/80371 (PMC12658395; doi:10.2196/80371)
Supplement: Multimedia Appendix 2 [file mental_v12i1e80371_app2.docx]

**Multimedia Appendix 2: Comparison of GPT citation outputs with actual citations**

| **Outcome** | **ChatGPT citation** | **Correct citation** |
| --- | --- | --- |
|  | **GENERAL REVIEW ON BINGE EATING DISORDER** |  |
| Non-fabricated but errors | Ágh, T., Kovács, G., Pawaskar, M., Supina, D., Inotai, A., & Vokó, Z. **(2016).** Epidemiology, health-related quality of life and economic burden of binge eating disorder: A systematic literature review. Eating and Weight Disorders, **21(3), 371–385.** [**https://doi.org/10.1007/s40519-016-0252-9**](https://doi.org/10.1007/s40519-016-0252-9) | Agh, T., Kovács, G., Pawaskar, M., Supina, D., Inotai, A., & Vokó, Z. **(2015).** Epidemiology, health-related quality of life and economic burden of binge eating disorder: a systematic literature review. Eating and Weight Disorders-Studies on Anorexia, Bulimia and Obesity, **20, 1-12.** [**https://doi.org/10.1007/s40519-014-0173-9**](https://doi.org/10.1007/s40519-014-0173-9) |
| Correct | American Psychiatric Association. (2013). Diagnostic and statistical manual of mental disorders (5th ed.). <https://doi.org/10.1176/appi.books.9780890425596> | American Psychiatric Association. (2013). Diagnostic and statistical manual of mental disorders (5th ed.). <https://doi.org/10.1176/appi.books.9780890425596> |
|  | Balodis, I. M., Kober, H., Worhunsky, P. D., White, M. A., Stevens, M. C., Pearlson, G. D., & Potenza, M. N. (2013). Monetary reward processing in obese individuals with and without binge eating disorder. Biological Psychiatry, 73(9), 877–886. <https://doi.org/10.1016/j.biopsych.2013.01.014> | Balodis, I. M., Kober, H., Worhunsky, P. D., White, M. A., Stevens, M. C., Pearlson, G. D., & Potenza, M. N. (2013). Monetary reward processing in obese individuals with and without binge eating disorder. Biological Psychiatry, 73(9), 877–886. <https://doi.org/10.1016/j.biopsych.2013.01.014> |
| Fabricated | Barakat, S., Maguire, S., Smith, K. E., & Park, R. J. (2019). Group and internet-based interventions for binge eating disorder: A systematic review and meta-analysis. Behaviour Research and Therapy, 123, 103515. <https://doi.org/10.1016/j.brat.2019.103515> | None found |
| Correct | Becker, C. B., Middlemass, K., Taylor, B., Johnson, C., & Gomez, F. (2017). Food insecurity and eating disorder pathology. International Journal of Eating Disorders, 50(9), 1031–1040. <https://doi.org/10.1002/eat.22735> | Becker, C. B., Middlemass, K., Taylor, B., Johnson, C., & Gomez, F. (2017). Food insecurity and eating disorder pathology. International Journal of Eating Disorders, 50(9), 1031–1040. <https://doi.org/10.1002/eat.22735> |
| Correct | Fairburn, C. G. (2008). Cognitive behavior therapy and eating disorders. Guilford Press. | Fairburn, C. G. (2008). Cognitive behavior therapy and eating disorders. Guilford Press. |
| Correct | Fairburn, C. G., Cooper, Z., & Shafran, R. (2003). Cognitive behaviour therapy for eating disorders: A "transdiagnostic" theory and treatment. Behaviour Research and Therapy, 41(5), 509–528. <https://doi.org/10.1016/S0005-7967(02)00088-8> | Fairburn, C. G., Cooper, Z., & Shafran, R. (2003). Cognitive behaviour therapy for eating disorders: A "transdiagnostic" theory and treatment. Behaviour Research and Therapy, 41(5), 509–528. <https://doi.org/10.1016/S0005-7967(02)00088-8> |
| Non-fabricated but errors | Fischer, S., Peterson, C., & McCarthy, D. (2013). A prospective test of the influence of negative urgency and expectancies on binge eating and purging. Psychology of Addictive Behaviors, 27(1), 294–300. [**https://doi.org/10.1037/a0027550**](https://doi.org/10.1037/a0027550) | Fischer, S., Peterson, C. M., & McCarthy, D. (2013). A prospective test of the influence of negative urgency and expectancies on binge eating and purging. *Psychology of Addictive Behaviors*, *27*(1), 294–300. [**https://doi.org/10.1037/a0029323**](https://doi.org/10.1037/a0029323) |
| Fabricated | Goode, R. W., Strickland, L. A., Brantley, P. J., & Appelhans, B. M. (2023). Addressing disparities in eating disorder treatment: A call to action. International Journal of Eating Disorders, 56(2), 170–175. <https://doi.org/10.1002/eat.23839> | None found |
| Fabricated | Grilo, C. M., White, M. A., & Masheb, R. M. (2009). Psychiatric comorbidity and behavioral treatment outcomes in binge eating disorder. Journal of Consulting and Clinical Psychology, 77(5), 915–925. <https://doi.org/10.1037/a0017064> | None found |
| Correct | Grucza, R. A., Przybeck, T. R., & Cloninger, C. R. (2007). Prevalence and correlates of binge eating disorder in a community sample. Comprehensive Psychiatry, 48(2), 124–131. <https://doi.org/10.1016/j.comppsych.2006.08.002> | Grucza, R. A., Przybeck, T. R., & Cloninger, C. R. (2007). Prevalence and correlates of binge eating disorder in a community sample. Comprehensive Psychiatry, 48(2), 124–131. <https://doi.org/10.1016/j.comppsych.2006.08.002> |
| Correct | Haedt-Matt, A. A., & Keel, P. K. (2011). Revisiting the affect regulation model of binge eating: A meta-analysis of studies using ecological momentary assessment. Psychological Bulletin, 137(4), 660–681. <https://doi.org/10.1037/a0023660> | Haedt-Matt, A. A., & Keel, P. K. (2011). Revisiting the affect regulation model of binge eating: A meta-analysis of studies using ecological momentary assessment. Psychological Bulletin, 137(4), 660–681. <https://doi.org/10.1037/a0023660> |
| Non-fabricated but errors | Hilbert, A., Bishop, M. E., Stein, R. I., Tanofsky-Kraff, M., Swenson, A. K., Welch, R. R., ... & Wilfley, D. E. (2012). Long-term efficacy of psychological treatments for binge eating disorder. British Journal of Psychiatry, 200(3), 232–237. [**https://doi.org/10.1192/bjp.bp.111.097808**](https://doi.org/10.1192/bjp.bp.111.097808) | Hilbert, A., Bishop, M. E., Stein, R. I., Tanofsky-Kraff, M., Swenson, A. K., Welch, R. R., & Wilfley, D. E. (2012). Long-term efficacy of psychological treatments for binge eating disorder. *The British Journal of Psychiatry*, *200*(3), 232–237. [**https://doi.org/10.1192/bjp.bp.110.089664**](https://doi.org/10.1192/bjp.bp.110.089664) |
| Fabricated | Hilbert, A., Petroff, D., Schmidt, R., Tuschen-Caffier, B., Vocks, S., & Herpertz, S. (2023). Efficacy of psychological treatments for binge eating disorder: A systematic review and network meta-analysis. The Lancet Psychiatry, 10(3), 198–209. <https://doi.org/10.1016/S2215-0366(22)00315-4> | None found |
| Correct | Hudson, J. I., Hiripi, E., Pope Jr, H. G., & Kessler, R. C. (2007). The prevalence and correlates of eating disorders in the National Comorbidity Survey Replication. Biological Psychiatry, 61(3), 348–358. <https://doi.org/10.1016/j.biopsych.2006.03.040> | Hudson, J. I., Hiripi, E., Pope Jr, H. G., & Kessler, R. C. (2007). The prevalence and correlates of eating disorders in the National Comorbidity Survey Replication. Biological Psychiatry, 61(3), 348–358. <https://doi.org/10.1016/j.biopsych.2006.03.040> |
| Fabricated | Johansson, T., Witteveen, A. B., Götestam Skorpen, C., Elstad, M., Rø, Ø., & Andreassen, O. A. (2020). A genome-wide association study of binge eating disorder. Translational Psychiatry, 10, 353. <https://doi.org/10.1038/s41398-020-01046-2> | None found |
| Non-fabricated but errors | Juarascio, A. S., Forman, E. M., & Herbert, J. D. **(2013).** Acceptance and commitment therapy versus cognitive therapy for the treatment of comorbid eating pathology. Behavior Modification, **37(6), 759–782.** [**https://doi.org/10.1177/0145445513486286**](https://doi.org/10.1177/0145445513486286) | Juarascio, A. S., Forman, E. M., & Herbert, J. D. **(2010).** Acceptance and Commitment Therapy versus Cognitive Therapy for the Treatment of Comorbid Eating Pathology. *Behavior Modification*, ***34*(2), 175–190.** <https://doi.org/10.1177/0145445510363472> |
| Correct | Kessler, R. C., Berglund, P. A., Chiu, W. T., Deitz, A. C., Hudson, J. I., Shahly, V., ... & Wilfley, D. E. (2013). The prevalence and correlates of binge eating disorder in the WHO World Mental Health Surveys. Biological Psychiatry, 73(9), 904–914. <https://doi.org/10.1016/j.biopsych.2012.11.020> | Kessler, R. C., Berglund, P. A., Chiu, W. T., Deitz, A. C., Hudson, J. I., Shahly, V., ... & Wilfley, D. E. (2013). The prevalence and correlates of binge eating disorder in the WHO World Mental Health Surveys. Biological Psychiatry, 73(9), 904–914. <https://doi.org/10.1016/j.biopsych.2012.11.020> |
| Correct | Kristeller, J. L., Wolever, R. Q., & Sheets, V. (2014). Mindfulness-based eating awareness training (MB-EAT) for binge eating: A randomized clinical trial. Mindfulness, 5(3), 282–297. <https://doi.org/10.1007/s12671-012-0179-1> | Kristeller, J., Wolever, R. Q., & Sheets, V. (2014). Mindfulness-Based Eating Awareness Training (MB-EAT) for binge eating: A randomized clinical trial. Mindfulness, 5(3), 282–297. <https://doi.org/10.1007/s12671-012-0179-1> |
| Non-fabricated but errors | Levine, M. P., & Piran, N**. (2019).** Reflections on the role of prevention in the link between sociocultural factors and eating disorders. Eating Disorders, **27(4), 366–383.** [**https://doi.org/10.1080/10640266.2019.1625314**](https://doi.org/10.1080/10640266.2019.1625314) | Levine, M. P., & Piran, N. **(2001).** Reflections on the role of prevention in the link between sociocultural factors and eating disorders. **Eat Disorders, 9, 261-5.** |
| Correct | Linardon, J., Wade, T. D., de la Piedad Garcia, X., & Brennan, L. (2017). The efficacy of cognitive-behavioral therapy for eating disorders: A systematic review and meta-analysis. Journal of Consulting and Clinical Psychology, 85(11), 1080–1094. <https://doi.org/10.1037/ccp0000245> | Linardon, J., Wade, T. D., de la Piedad Garcia, X., & Brennan, L. (2017). The efficacy of cognitive-behavioral therapy for eating disorders: A systematic review and meta-analysis. Journal of Consulting and Clinical Psychology, 85(11), 1080–1094. <https://doi.org/10.1037/ccp0000245> |
| Non-fabricated but errors | McElroy, S. L., Guerdjikova, A. I., Mori, N., & O'Melia, A. M. **(2015).** Current pharmacotherapy options for binge eating disorder. Expert Opinion on Pharmacotherapy, **16(10), 1463–1478.** [**https://doi.org/10.1517/14656566.2015.1046837**](https://doi.org/10.1517/14656566.2015.1046837) | McElroy, S. L., Guerdjikova, A. I., Mori, N., & O’Melia, A. M. **(2012).** Current pharmacotherapy options for bulimia nervosa and binge eating disorder. *Expert Opinion on Pharmacotherapy*, ***13*(14), 2015–2026.** [**https://doi.org/10.1517/14656566.2012.721781**](https://doi.org/10.1517/14656566.2012.721781) |
| Non-fabricated but errors | Mitchell, J. E., Devlin, M. J., de Zwaan, M., Crow, S. J., & Peterson, C. B. **(2013).** Binge eating disorder: Clinical foundations and treatment. Guilford Press. | Mitchell, J. E., Devlin, M. J., de Zwaan, M., Crow, S. J., & Peterson, C. B. (**2008).** *Binge-eating disorder: Clinical foundations and treatment*. Guilford Press. |
| Non-fabricated but errors | Monteleone, P., Di Lieto, A., Tortorella, A., Longobardi, N., & Maj, M. **(2012). Circulating levels of leptin and ghrelin in binge-eating disorder: Relationship with binge-eating episodes and psychopathological features. Psychological Medicine, 42(8), 1839–1846.** [**https://doi.org/10.1017/S0033291711002766**](https://doi.org/10.1017/S0033291711002766) | Monteleone, P., Di Lieto, A., Tortorella, A., Longobardi, N., & Maj, M. **(2000).** **Circulating leptin in patients with anorexia nervosa, bulimia nervosa or binge-eating disorder: relationship to body weight, eating patterns, psychopathology and endocrine changes.** ***Psychiatry research*,** ***94*(2), 121-129. https://doi.org/10.1016/S0165-1781(00)00144-X** |
| Non-fabricated but errors | **Rodgers, R. F., Donovan, E., Cousineau, T., Yates, K., McGowan, K., Cook, E., ... & Lukowicz, M. (2016). Instagram use and young women's body image concerns and self-objectification: Testing mediational pathways**. New Media & Society, 20(4), 1380–1395. [**https://doi.org/10.1177/1461444817694499**](https://doi.org/10.1177/1461444817694499) | **Fardouly, J., Willburger, B. K., & Vartanian, L. R. (2018).** **Instagram use and young women’s body image concerns and self-objectification: Testing mediational pathways.** *New Media & Society*, *20*(4), 1380–1395. [**https://doi.org/10.1177/1461444817694499**](https://doi.org/10.1177/1461444817694499) |
| Fabricated | Ranzenhofer, L. M., Engel, S. G., Cohen, L. A., et al. (2014). Behavioral and environmental correlates of binge eating in children and adolescents: A review. Eating Behaviors, 15(4), 497–505. <https://doi.org/10.1016/j.eatbeh.2014.07.003> | None found |
| Correct | Safer, D. L., Telch, C. F., & Agras, W. S. (2001). Dialectical behavior therapy for bulimia nervosa. American Journal of Psychiatry, 158(4), 632–634. <https://doi.org/10.1176/appi.ajp.158.4.632> | Safer, D. L., Telch, C. F., & Agras, W. S. (2001). Dialectical behavior therapy for bulimia nervosa. American Journal of Psychiatry, 158(4), 632–634. <https://doi.org/10.1176/appi.ajp.158.4.632> |
| Non-fabricated but errors | Schienle, A., Schäfer, A., Hermann, A., & Vaitl, D. **(2016).** Binge-eating disorder: Reward sensitivity and brain activation to images of food. Biological Psychology, **120, 130–136.** [**https://doi.org/10.1016/j.biopsycho.2016.09.004**](https://doi.org/10.1016/j.biopsycho.2016.09.004) | Schienle, A., Schäfer, A., Hermann, A., & Vaitl, D. **(2009).** Binge-eating disorder: Reward sensitivity and brain activation to images of food. *Biological Psychiatry*, ***65*(8), 654–661.** [**https://doi.org/10.1016/j.biopsych.2008.09.028**](https://doi.org/10.1016/j.biopsych.2008.09.028) |
| Non-fabricated but errors | **Streatfeild, J., Hickson, J., Austin, S. B., Hutcheson, R., Kandel, K. J., Lampert, J. G., ... & Le Grange, D.** (2021). Social and economic cost of eating disorders in the United States: Evidence to inform policy action. International Journal of Eating Disorders, 54(5), 851–868. <https://doi.org/10.1002/eat.23486> | **Streatfeild, J., Hickson, J., Austin, S. B., Hutcheson, R., Kandel, J. S., Lampert, J. G., Myers, E. M., Richmond, T. K., Samnaliev, M., Velasquez, K., Weissman, R. S., & Pezzullo, L. (2021).** Social and economic cost of eating disorders in the United States: Evidence to inform policy action. *International Journal of Eating Disorders*, *54*(5), 851–868. <https://doi.org/10.1002/eat.23486> |
| Correct | Telch, C. F., Agras, W. S., & Linehan, M. M. (2001). Dialectical behavior therapy for binge eating disorder. Journal of Consulting and Clinical Psychology, 69(6), 1061–1065. <https://doi.org/10.1037/0022-006X.69.6.1061> | Telch, C. F., Agras, W. S., & Linehan, M. M. (2001). Dialectical behavior therapy for binge eating disorder. Journal of Consulting and Clinical Psychology, 69(6), 1061–1065. <https://doi.org/10.1037/0022-006X.69.6.1061> |
| Correct | Trace, S. E., Baker, J. H., Penas-Lledo, E., & Bulik, C. M. (2013). The genetics of eating disorders. Annual Review of Clinical Psychology, 9, 589–620. <https://doi.org/10.1146/annurev-clinpsy-050212-185546> | Trace, S. E., Baker, J. H., Penas-Lledo, E., & Bulik, C. M. (2013). The genetics of eating disorders. Annual Review of Clinical Psychology, 9, 589–620. <https://doi.org/10.1146/annurev-clinpsy-050212-185546> |
| Correct | Udo, T., & Grilo, C. M. (2018). Prevalence and correlates of DSM-5-defined eating disorders in a nationally representative sample of U.S. adults. Biological Psychiatry, 84(5), 345–354. <https://doi.org/10.1016/j.biopsych.2018.03.014> | Udo, T., & Grilo, C. M. (2018). Prevalence and correlates of DSM-5-defined eating disorders in a nationally representative sample of U.S. adults. Biological Psychiatry, 84(5), 345–354. <https://doi.org/10.1016/j.biopsych.2018.03.014> |
| Correct | Whiteside, U., Chen, E., Neighbors, C., Hunter, D., Lo, T., & Larimer, M. (2007). Difficulties regulating emotions: Do binge eaters have fewer strategies to modulate and tolerate negative affect? Eating Behaviors, 8(2), 162–169. <https://doi.org/10.1016/j.eatbeh.2006.04.001> | Whiteside, U., Chen, E., Neighbors, C., Hunter, D., Lo, T., & Larimer, M. (2007). Difficulties regulating emotions: Do binge eaters have fewer strategies to modulate and tolerate negative affect? Eating Behaviors, 8(2), 162–169. <https://doi.org/10.1016/j.eatbeh.2006.04.001> |
| Correct | Wilfley, D. E., Welch, R. R., Stein, R. I., Spurrell, E. B., Cohen, L. R., Saelens, B. E., ... & Matt, G. E. (2002). A randomized comparison of group cognitive-behavioral therapy and group interpersonal psychotherapy for the treatment of overweight individuals with binge-eating disorder. Archives of General Psychiatry, 59(8), 713–721. <https://doi.org/10.1001/archpsyc.59.8.713> | Wilfley, D. E., Welch, R. R., Stein, R. I., Spurrell, E. B., Cohen, L. R., Saelens, B. E., ... & Matt, G. E. (2002). A randomized comparison of group cognitive-behavioral therapy and group interpersonal psychotherapy for the treatment of overweight individuals with binge-eating disorder. Archives of General Psychiatry, 59(8), 713–721. <https://doi.org/10.1001/archpsyc.59.8.713> |
| Correct | Wilson, G. T., Wilfley, D. E., Agras, W. S., & Bryson, S. W. (2010). Psychological treatments of binge eating disorder. Archives of General Psychiatry, 67(1), 94–101. <https://doi.org/10.1001/archgenpsychiatry.2009.170> | Wilson, G. T., Wilfley, D. E., Agras, W. S., & Bryson, S. W. (2010). Psychological treatments of binge eating disorder. Archives of General Psychiatry, 67(1), 94–101. <https://doi.org/10.1001/archgenpsychiatry.2009.170> |
| Non-fabricated but errors | **Yilmaz, Z., Gottfried, H., Jennings, K. M., & Hardaway, A. (2019). Childhood maltreatment and eating disorders: A systematic review and meta-analysis**. Psychological Medicine, 49(9), **1403–1415.** [**https://doi.org/10.1017/S0033291718003825**](https://doi.org/10.1017/S0033291718003825) | **Molendijk, M. L., Hoek, H. W., Brewerton, T. D., & Elzinga, B. M. (2017). Childhood maltreatment and eating disorder pathology: A systematic review and dose-response meta-analysis.** *Psychological Medicine*, ***47*(8), 1402–1416.** **https://doi.org/10.1017/S0033291716003561** |
|  | **SPECIALIZED REVIEW ON BINGE EATING DISORDER** |  |
| Non-fabricated but errors | Aardoom, J. J., Dingemans, A. E., Spinhoven, P., & van Furth, E. F. **(2016).** Treating eating disorders over the internet: A systematic review and future research directions. *International Journal of Eating Disorders,* ***49*(2), 103–112.** [**https://doi.org/10.1002/eat.22445**](https://doi.org/10.1002/eat.22445) | Aardoom, J. J., Dingemans, A. E., Spinhoven, P., & Furth, E. F. **(2013).** Treating eating disorders over the internet: A systematic review and future research directions. *International Journal of Eating Disorders*, ***46*(6), 539–552. https://doi.org/10.1002/eat.22135** |
| Correct | American Psychiatric Association. (2013). *Diagnostic and statistical manual of mental disorders* (5th ed.). <https://doi.org/10.1176/appi.books.9780890425596> | American Psychiatric Association. (2013). *Diagnostic and statistical manual of mental disorders* (5th ed.). <https://doi.org/10.1176/appi.books.9780890425596> |
| Correct | Baumel, A., Muench, F., Edan, S., & Kane, J. M. (2019). Objective user engagement with mental health apps: Systematic search and panel-based usage analysis. *Journal of Medical Internet Research, 21*(9), e14567. <https://doi.org/10.2196/14567> | Baumel, A., Muench, F., Edan, S., & Kane, J. M. (2019). Objective user engagement with mental health apps: Systematic search and panel-based usage analysis. *Journal of Medical Internet Research, 21*(9), e14567. <https://doi.org/10.2196/14567> |
| Non-fabricated but errors | Beintner, I., Jacobi, C., & Taylor, C. B. (2014). Effects of an internet-based prevention programme for eating disorders in the USA and Germany—A meta-analytic review. *European Eating Disorders Review,* ***22*(5), 329–341.** [**https://doi.org/10.1002/erv.2300**](https://doi.org/10.1002/erv.2300) | Beintner, I., Jacobi, C., & Taylor, C. B. **(2012).** Effects of an Internet‐based prevention programme for eating disorders in the USA and Germany — A meta‐analytic review. *European Eating Disorders Review*, ***20*(1), 1–8. https://doi.org/10.1002/erv.1130** |
| Fabricated | Bennett, B. L., Goldstein, C. M., Gathright, E. C., Hughes, J. W., & Latner, J. D. (2019). Outcomes of a smartphone-based guided self-help intervention for binge eating: A randomized controlled trial. *Appetite, 141*, 104317. <https://doi.org/10.1016/j.appet.2019.104317> | None found |
| Correct | Carrard, I., Crépin, C., Rouget, P., Lam, T., Golay, A., & Van der Linden, M. (2011). Randomised controlled trial of a guided self-help treatment on the internet for binge eating disorder. *Behaviour Research and Therapy, 49*(8), 482–491. https//doi.org/10.1016/j.brat.2011.05.004 | Carrard, I., Crépin, C., Rouget, P., Lam, T., Golay, A., & Van der Linden, M. (2011). Randomised controlled trial of a guided self-help treatment on the internet for binge eating disorder. *Behaviour Research and Therapy, 49*(8), 482–491. https//doi.org/10.1016/j.brat.2011.05.004 |
| Fabricated | Duarte, C., Ferreira, C., & Pinto‐Gouveia, J. (2017). Effectiveness of a mindfulness‐based intervention on body image and shame in women with binge eating disorder. *Mindfulness, 8*(3), 715–727. <https://doi.org/10.1007/s12671-016-0644-1> | None found |
| Fabricated | Grilo, C. M., White, M. A., & Masheb, R. M. (2013). DSM-5 diagnostic criteria for binge eating disorder: A test of the reliability and validity in a clinical sample. *Journal of Consulting and Clinical Psychology, 81*(1), 123–128. <https://doi.org/10.1037/a0030843> | None found |
| Non-fabricated but errors | Hilbert, A., Bishop, M. E., Stein, R. I., Tanofsky-Kraff, M., Swenson, A. K., Welch, R. R., & Wilfley, D. E. (2012). Long-term efficacy of psychological treatments for binge eating disorder. *British Journal of Psychiatry, 200*(3), 232–237. [**https://doi.org/10.1192/bjp.bp.111.101796**](https://doi.org/10.1192/bjp.bp.111.101796) | Hilbert, A., Bishop, M. E., Stein, R. I., Tanofsky-Kraff, M., Swenson, A. K., Welch, R. R., & Wilfley, D. E. (2012). Long-term efficacy of psychological treatments for binge eating disorder. *The British Journal of Psychiatry*, *200*(3), 232–237. [**https://doi.org/10.1192/bjp.bp.110.089664**](https://doi.org/10.1192/bjp.bp.110.089664) |
| Correct | Hilvert-Bruce, Z., Rossouw, P. J., Wong, N., Sunderland, M., & Andrews, G. (2012). Adherence as a determinant of effectiveness of internet cognitive behavioural therapy for anxiety and depressive disorders. *Behaviour Research and Therapy, 50*(7–8), 463–468. <https://doi.org/10.1016/j.brat.2012.04.001> | Hilvert-Bruce, Z., Rossouw, P. J., Wong, N., Sunderland, M., & Andrews, G. (2012). Adherence as a determinant of effectiveness of internet cognitive behavioural therapy for anxiety and depressive disorders. *Behaviour Research and Therapy, 50*(7–8), 463–468. <https://doi.org/10.1016/j.brat.2012.04.001> |
| Correct | Kazdin, A. E., & Rabbitt, S. M. (2013). Novel models for delivering mental health services and reducing the burdens of mental illness. *Clinical Psychological Science, 1*(2), 170–191. <https://doi.org/10.1177/2167702612463566> | Kazdin, A. E., & Rabbitt, S. M. (2013). Novel models for delivering mental health services and reducing the burdens of mental illness. *Clinical Psychological Science, 1*(2), 170–191. <https://doi.org/10.1177/2167702612463566> |
| Correct | Kessler, R. C., Berglund, P. A., Chiu, W. T., et al. (2013). The prevalence and correlates of binge eating disorder in the WHO World Mental Health Surveys. *Biological Psychiatry, 73*(9), 904–914. <https://doi.org/10.1016/j.biopsych.2012.11.020> | Kessler, R. C., Berglund, P. A., Chiu, W. T., et al. (2013). The prevalence and correlates of binge eating disorder in the WHO World Mental Health Surveys. *Biological Psychiatry, 73*(9), 904–914. <https://doi.org/10.1016/j.biopsych.2012.11.020> |
| Correct | Linardon, J., & Fuller-Tyszkiewicz, M. (2020). Attrition and adherence in smartphone-delivered interventions for mental health problems: A systematic and meta-analytic review. *Journal of Consulting and Clinical Psychology, 88*(1), 1–13. <https://doi.org/10.1037/ccp0000459> | Linardon, J., & Fuller-Tyszkiewicz, M. (2020). Attrition and adherence in smartphone-delivered interventions for mental health problems: A systematic and meta-analytic review. *Journal of Consulting and Clinical Psychology, 88*(1), 1–13. <https://doi.org/10.1037/ccp0000459> |
| Fabricated | Linardon, J., Messer, M. (2019). Smartphone-delivered cognitive behavioral therapy for eating disorder symptoms: A randomized controlled trial. *Psychotherapy and Psychosomatics, 88*(1), 28–36. <https://doi.org/10.1159/000495044> | None found |
| Fabricated | Linardon, J., Shatte, A., & Messer, M. (2022). Mobile app–delivered cognitive behavior therapy for binge eating disorder: A randomized controlled trial. *Behaviour Research and Therapy, 153*, 104089. <https://doi.org/10.1016/j.brat.2022.104089> | None found |
| Fabricated | Linardon, J., Shatte, A., Rosato, J., & Fuller-Tyszkiewicz, M. (2020). Efficacy of digital interventions for eating disorder symptoms: A meta-analysis of randomized controlled trials. *Clinical Psychology Review, 80*, 101899. <https://doi.org/10.1016/j.cpr.2020.101899> | None found |
| Fabricated | Sahlan, R. N., Farrell, N. R., & Thompson-Brenner, H. (2022). Self-compassion and emotion regulation in binge eating disorder. *Appetite, 170*, 105899. <https://doi.org/10.1016/j.appet.2021.105899> | None found |
| Fabricated | Schleider, J. L., Dobias, M., & Pati, S. (2021). Single-session interventions for adolescent mental health: A meta-analysis. *Clinical Psychology Review, 83*, 101944. <https://doi.org/10.1016/j.cpr.2020.101944> | None found |
| Fabricated | Terp, H., Laursen, B. S., Jørgensen, R., Mainz, J., & Mainz, H. (2021). Web-based interventions for adults with severe mental illness: A scoping review. *International Journal of Mental Health Nursing, 30*(1), 15–33. <https://doi.org/10.1111/inm.12780> | None found |
| Non-fabricated but errors | Tregarthen, J. P., Lock, J., & Darcy, A. M. (2019). **Development of a smartphone application for eating disorder recovery**. *International Journal of Eating Disorders,* ***52*(9), 1071–1080. https://doi.org/10.1002/eat.23136** | Tregarthen, J. P., Lock, J., & Darcy, A. M. **(2015).** **Development of a smartphone application for eating disorder self-monitoring**. *International Journal of Eating Disorders*, ***48*(7), 972–982. https://doi.org/10.1002/eat.22386** |
| Correct | Udo, T., & Grilo, C. M. (2018). Prevalence and correlates of DSM-5–defined eating disorders in a nationally representative sample of US adults. *Biological Psychiatry, 84*(5), 345–354. <https://doi.org/10.1016/j.biopsych.2018.03.014> | Udo, T., & Grilo, C. M. (2018). Prevalence and correlates of DSM-5–defined eating disorders in a nationally representative sample of US adults. *Biological Psychiatry, 84*(5), 345–354. <https://doi.org/10.1016/j.biopsych.2018.03.014> |
| Non-fabricated but errors | Wagner, B., Nagl, M., Dölemeyer, R., Klinitzke, G., Steinig, J., Hilbert, A., & Kersting, A. (2016). Randomized controlled trial of an internet-based cognitive–behavioral treatment program for binge-eating disorder. *Behavior Therapy, 47*(4), 500–514**.** [**https://doi.org/10.1016/j.beth.2016.03.002**](https://doi.org/10.1016/j.beth.2016.03.002) | Wagner, B., Nagl, M., Dölemeyer, R., Klinitzke, G., Steinig, J., Hilbert, A., & Kersting, A. (2016). Randomized controlled trial of an internet-based cognitive-behavioral treatment program for binge-eating disorder. *Behavior Therapy*, *47*(4), 500–514. [**https://doi.org/10.1016/j.beth.2016.01.006**](https://doi.org/10.1016/j.beth.2016.01.006) |
| Fabricated | Yim, H. W., Bae, K., Choi, Y., et al. (2020). User acceptance of a mobile app for early mental health intervention in college students. *Journal of Medical Internet Research, 22*(6), e15465. <https://doi.org/10.2196/15465> | None found |
| Fabricated | Zucker, N., Taylor, C. B., Bulik, C. M., & Fairburn, C. G. (2021). Data-driven health technology interventions for eating disorders. *Psychiatric Clinics, 44*(4), 525–536. <https://doi.org/10.1016/j.psc.2021.07.008> | None found |
|  | **GENERAL REVIEW ON MAJOR DEPRESSIVE DISORDER** |  |
| Correct | A-Tjak, J. G. L., Davis, M. L., Morina, N., Powers, M. B., Smits, J. A. J., & Emmelkamp, P. M. G. (2015). A meta-analysis of the efficacy of acceptance and commitment therapy for clinically relevant mental and physical health problems. *Psychotherapy and Psychosomatics, 84*(1), 30–36. <https://doi.org/10.1159/000365764> | A-Tjak, J. G. L., Davis, M. L., Morina, N., Powers, M. B., Smits, J. A. J., & Emmelkamp, P. M. G. (2015). A meta-analysis of the efficacy of acceptance and commitment therapy for clinically relevant mental and physical health problems. *Psychotherapy and Psychosomatics, 84*(1), 30–36. <https://doi.org/10.1159/000365764> |
| Correct | Andersson, G., Carlbring, P., Titov, N., & Lindefors, N. (2019). Internet interventions for adults with anxiety and mood disorders: A narrative umbrella review of recent meta-analyses. *The Canadian Journal of Psychiatry, 64*(7), 465–470. <https://doi.org/10.1177/0706743719839381> | Andersson, G., Carlbring, P., Titov, N., & Lindefors, N. (2019). Internet interventions for adults with anxiety and mood disorders: A narrative umbrella review of recent meta-analyses. *The Canadian Journal of Psychiatry, 64*(7), 465–470. <https://doi.org/10.1177/0706743719839381> |
| Non-fabricated but errors | Beck, J. S. **(2008).** *Cognitive behavior therapy: Basics and beyond* (2nd ed.). Guilford Press. | Beck, J. S. **(2011).** Cognitive behavior therapy: Basics and beyond (2nd ed.). Guilford Press. |
| Correct | Berking, M., Wirtz, C. M., Svaldi, J., & Hofmann, S. G. (2014). Emotion regulation predicts symptoms of depression over five years. *Behaviour Research and Therapy, 57*, 13–20. <https://doi.org/10.1016/j.brat.2014.03.003> | Berking, M., Wirtz, C. M., Svaldi, J., & Hofmann, S. G. (2014). Emotion regulation predicts symptoms of depression over five years. *Behaviour Research and Therapy, 57*, 13–20. <https://doi.org/10.1016/j.brat.2014.03.003> |
| Correct | Bromet, E., Andrade, L. H., Hwang, I., Sampson, N. A., Alonso, J., de Girolamo, G., ... & Kessler, R. C. (2011). Cross-national epidemiology of DSM-IV major depressive episode. *BMC Medicine, 9(1)*, 90. <https://doi.org/10.1186/1741-7015-9-90> | Bromet, E., Andrade, L. H., Hwang, I., Sampson, N. A., Alonso, J., de Girolamo, G., de Graaf, R., Demyttenaere, K., Hu, C., Iwata, N., Karam, A. N., Kaur, J., Kostyuchenko, S., Lépine, J.-P., Levinson, D., Matschinger, H., Mora, M. E. M., Browne, M. O., Posada-Villa, J., & Viana, M. C., Williams, D. R., Kessler, R. C. (2011). Cross-national epidemiology of DSM-IV major depressive episode. BMC Medicine, 9(1), 90. <https://doi.org/10.1186/1741-7015-9-90> |
| Correct | Butler, A. C., Chapman, J. E., Forman, E. M., & Beck, A. T. (2006). The empirical status of cognitive-behavioral therapy: A review of meta-analyses. *Clinical Psychology Review, 26*(1), 17–31. <https://doi.org/10.1016/j.cpr.2005.07.003> | Butler, A. C., Chapman, J. E., Forman, E. M., & Beck, A. T. (2006). The empirical status of cognitive-behavioral therapy: A review of meta-analyses. *Clinical Psychology Review, 26*(1), 17–31. <https://doi.org/10.1016/j.cpr.2005.07.003> |
| Non-fabricated but errors | Cacioppo, J. T., Hughes, M. E., Waite, L. J., Hawkley, L. C., & Thisted, R. A. **(2010).** Loneliness as a specific risk factor for depressive symptoms: Cross-sectional and longitudinal analyses. *Psychology and Aging,* ***25*(2), 453–463.** [**https://doi.org/10.1037/a0017216**](https://doi.org/10.1037/a0017216) | Cacioppo, J. T., Hughes, M. E., Waite, L. J., Hawkley, L. C., & Thisted, R. A. (**2006).** Loneliness as a specific risk factor for depressive symptoms: Cross-sectional and longitudinal analyses. Psychology and Aging, **21(1), 140–151.** [**https://doi.org/10.1037/0882-7974.21.1.140**](https://doi.org/10.1037/0882-7974.21.1.140) |
| Non-fabricated but errors | **Cuijpers, P., Karyotaki, E., de Wit, L., Ebert, D. D., & Klein, J. P. (2020).** The effects of psychotherapies for major depression in adults on remission, recovery and improvement: A meta-analysis. *Journal of Affective Disorders,* ***277*, 142–149.** [**https://doi.org/10.1016/j.jad.2020.08.057**](https://doi.org/10.1016/j.jad.2020.08.057) | **Cuijpers, P., Karyotaki, E., Weitz, E., Andersson, G., Hollon, S. D., & van Straten, A. (2014).** The effects of psychotherapies for major depression in adults on remission, recovery and improvement: A meta-analysis. Journal of Affective Disorders, **159, 118–126.** [**https://doi.org/10.1016/j.jad.2014.02.026**](https://doi.org/10.1016/j.jad.2014.02.026) |
| Non-fabricated but errors | **Cuijpers, P., Karyotaki, E., Weitz, E., Andersson, G., Hollon, S. D., van Straten, A., & Ebert, D. D. (2016).** The effects of psychotherapies for major depression in adults on remission, recovery and improvement: A meta-analysis. ***Journal of Affective Disorders, 202*, 511–517.** [**https://doi.org/10.1016/j.jad.2016.05.050**](https://doi.org/10.1016/j.jad.2016.05.050) | **Cuijpers, P., Karyotaki, E., Weitz, E., Andersson, G., Hollon, S. D., & van Straten, A. (2014).** The effects of psychotherapies for major depression in adults on remission, recovery and improvement: A meta-analysis. Journal of Affective Disorders, **159, 118–126. https://doi.org/10.1016/j.jad.2014.02.026** |
| Correct | Cuijpers, P., Quero, S., Dowrick, C., & Arroll, B. (2019). Psychological treatment of depression in primary care: Recent developments. *Current Psychiatry Reports, 21*, 129. <https://doi.org/10.1007/s11920-019-1117-x> | Cuijpers, P., Quero, S., Dowrick, C., & Arroll, B. (2019). Psychological treatment of depression in primary care: Recent developments. *Current Psychiatry Reports, 21*, 129. <https://doi.org/10.1007/s11920-019-1117-x> |
| Non-fabricated but errors | Cuijpers, P., Noma, H., Karyotaki, E., Cipriani, A., & Furukawa, T. A. **(2021).** Effectiveness and acceptability of cognitive behavior therapy delivery formats in adults with depression: A network meta-analysis. *JAMA Psychiatry,* ***78*(**7), **700–708.** [**https://doi.org/10.1001/jamapsychiatry.2021.0310**](https://doi.org/10.1001/jamapsychiatry.2021.0310) | Cuijpers, P., Noma, H., Karyotaki, E., Cipriani, A., & Furukawa, T. A. **(2019).** Effectiveness and acceptability of cognitive behavior therapy delivery formats in adults with depression: A network meta-analysis. JAMA Psychiatry, **76**(7), **700–707. https://doi.org/10.1001/jamapsychiatry.2019.0268** |
| Correct | Disner, S. G., Beevers, C. G., Haigh, E. A. P., & Beck, A. T. (2011). Neural mechanisms of the cognitive model of depression. *Nature Reviews Neuroscience, 12*(8), 467–477. <https://doi.org/10.1038/nrn3027> | Disner, S. G., Beevers, C. G., Haigh, E. A. P., & Beck, A. T. (2011). Neural mechanisms of the cognitive model of depression. *Nature Reviews Neuroscience, 12*(8), 467–477. <https://doi.org/10.1038/nrn3027> |
| Correct | Ekers, D., Webster, L., Van Straten, A., Cuijpers, P., Richards, D., & Gilbody, S. (2014). Behavioural activation for depression; An update of meta-analysis of effectiveness and sub group analysis. *PLOS ONE, 9*(6), e100100. <https://doi.org/10.1371/journal.pone.0100100> | Ekers, D., Webster, L., Van Straten, A., Cuijpers, P., Richards, D., & Gilbody, S. (2014). Behavioural activation for depression; An update of meta-analysis of effectiveness and sub group analysis. *PLOS ONE, 9*(6), e100100. <https://doi.org/10.1371/journal.pone.0100100> |
| Correct | GBD 2019 Mental Disorders Collaborators. (2022). Global, regional, and national burden of 12 mental disorders in 204 countries and territories, 1990–2019: A systematic analysis for the Global Burden of Disease Study 2019. *The Lancet Psychiatry, 9*(2), 137–150. <https://doi.org/10.1016/S2215-0366(21)00395-3> | GBD 2019 Mental Disorders Collaborators. (2022). Global, regional, and national burden of 12 mental disorders in 204 countries and territories, 1990–2019: A systematic analysis for the Global Burden of Disease Study 2019. *The Lancet Psychiatry, 9*(2), 137–150. <https://doi.org/10.1016/S2215-0366(21)00395-3> |
| Correct | Goodman, S. H., Rouse, M. H., Connell, A. M., Broth, M. R., Hall, C. M., & Heyward, D. (2011). Maternal depression and child psychopathology: A meta-analytic review. *Clinical Child and Family Psychology Review, 14*, 1–27. <https://doi.org/10.1007/s10567-010-0080-1> | Goodman, S. H., Rouse, M. H., Connell, A. M., Broth, M. R., Hall, C. M., & Heyward, D. (2011). Maternal depression and child psychopathology: A meta-analytic review. *Clinical Child and Family Psychology Review, 14*, 1–27. <https://doi.org/10.1007/s10567-010-0080-1> |
| Non-fabricated but errors | **Greenberg, P. E., Fournier, A**. **A., Sisitsky, T., Pike, C. T., & Kessler, R. C**. (2021). The economic burden of adults with major depressive disorder in the United States (2010 and 2018). *Pharmacoeconomics, 39*(6), 653–665. <https://doi.org/10.1007/s40273-021-01019-4> | **Greenberg, P. E., Fournier, A.-A., Sisitsky, T., Simes, M., Berman, R., Koenigsberg, S. H., & Kessler, R. C.** (2021). The Economic Burden of Adults with Major Depressive Disorder in the United States (2010 and 2018). PharmacoEconomics, 39(6), 653–665. <https://doi.org/10.1007/s40273-021-01019-4> |
| Correct | Heim, C., & Nemeroff, C. B. (2001). The role of childhood trauma in the neurobiology of mood and anxiety disorders: Preclinical and clinical studies. *Biological Psychiatry, 49*(12), 1023–1039. <https://doi.org/10.1016/S0006-3223(01)01157-X> | Heim, C., & Nemeroff, C. B. (2001). The role of childhood trauma in the neurobiology of mood and anxiety disorders: Preclinical and clinical studies. *Biological Psychiatry, 49*(12), 1023–1039. <https://doi.org/10.1016/S0006-3223(01)01157-X> |
| Correct | Hollon, S. D., Stewart, M. O., & Strunk, D. (2006). Enduring effects for cognitive behavior therapy in the treatment of depression and anxiety. *Annual Review of Psychology, 57*(1), 285–315. <https://doi.org/10.1146/annurev.psych.57.102904.190044> | Hollon, S. D., Stewart, M. O., & Strunk, D. (2006). Enduring effects for cognitive behavior therapy in the treatment of depression and anxiety. *Annual Review of Psychology, 57*(1), 285–315. <https://doi.org/10.1146/annurev.psych.57.102904.190044> |
| Correct | Howard, D. M., Adams, M. J., Clarke, T. K., Hafferty, J. D., Gibson, J., Shirali, M., ... & McIntosh, A. M. (2019). Genome-wide meta-analysis of depression identifies 102 independent variants and highlights the importance of the prefrontal brain regions. *Nature Neuroscience, 22*(3), 343–352. <https://doi.org/10.1038/s41593-018-0326-7> | Howard, D. M., Adams, M. J., Clarke, T. K., Hafferty, J. D., Gibson, J., Shirali, M., ... & McIntosh, A. M. (2019). Genome-wide meta-analysis of depression identifies 102 independent variants and highlights the importance of the prefrontal brain regions. *Nature Neuroscience, 22*(3), 343–352. <https://doi.org/10.1038/s41593-018-0326-7> |
| Correct | Karyotaki, E., Efthimiou, O., Miguel, C., Bermpohl, F. M. G., Furukawa, T. A., Cuijpers, P., ... & Ebert, D. D. (2021). Internet-based cognitive behavioral therapy for depression: A systematic review and individual patient data network meta-analysis. *JAMA Psychiatry, 78*(4), 361–371. <https://doi.org/10.1001/jamapsychiatry.2020.4364> | Karyotaki, E., Efthimiou, O., Miguel, C., Bermpohl, F. M. G., Furukawa, T. A., Cuijpers, P., ... & Ebert, D. D. (2021). Internet-based cognitive behavioral therapy for depression: A systematic review and individual patient data network meta-analysis. *JAMA Psychiatry, 78*(4), 361–371. <https://doi.org/10.1001/jamapsychiatry.2020.4364> |
| Correct | Kendler, K. S., Karkowski, L. M., & Prescott, C. A. (1999). Causal relationship between stressful life events and the onset of major depression. *The American Journal of Psychiatry, 156*(6), 837–841. <https://doi.org/10.1176/ajp.156.6.837> | Kendler, K. S., Karkowski, L. M., & Prescott, C. A. (1999). Causal relationship between stressful life events and the onset of major depression. *The American Journal of Psychiatry, 156*(6), 837–841. <https://doi.org/10.1176/ajp.156.6.837> |
| Correct | Kiecolt-Glaser, J. K., Derry, H. M., & Fagundes, C. P. (2015). Inflammation: Depression fans the flames and feasts on the heat. *American Journal of Psychiatry, 172*(11), 1075–1091. <https://doi.org/10.1176/appi.ajp.2015.15020152> | Kiecolt-Glaser, J. K., Derry, H. M., & Fagundes, C. P. (2015). Inflammation: Depression fans the flames and feasts on the heat. *American Journal of Psychiatry, 172*(11), 1075–1091. <https://doi.org/10.1176/appi.ajp.2015.15020152> |
| Correct | Kotov, R., Gamez, W., Schmidt, F., & Watson, D. (2010). Linking “big” personality traits to anxiety, depressive, and substance use disorders: A meta-analysis. *Psychological Bulletin, 136*(5), 768–821. <https://doi.org/10.1037/a0020327> | Kotov, R., Gamez, W., Schmidt, F., & Watson, D. (2010). Linking “big” personality traits to anxiety, depressive, and substance use disorders: A meta-analysis. *Psychological Bulletin, 136*(5), 768–821. <https://doi.org/10.1037/a0020327> |
| Correct | Kuyken, W., Warren, F., Taylor, R. S., Whalley, B., Crane, C., Bondolfi, G., ... & Segal, Z. (2016). Efficacy of mindfulness-based cognitive therapy in prevention of depressive relapse: An individual patient data meta-analysis from randomized trials. *JAMA Psychiatry, 73*(6), 565–574. <https://doi.org/10.1001/jamapsychiatry.2016.0076> | Kuyken, W., Warren, F., Taylor, R. S., Whalley, B., Crane, C., Bondolfi, G., ... & Segal, Z. (2016). Efficacy of mindfulness-based cognitive therapy in prevention of depressive relapse: An individual patient data meta-analysis from randomized trials. *JAMA Psychiatry, 73*(6), 565–574. <https://doi.org/10.1001/jamapsychiatry.2016.0076> |
| Correct | Lepine, J. P., & Briley, M. (2011). The increasing burden of depression. *Neuropsychiatric Disease and Treatment, 7*(Suppl 1), 3–7. <https://doi.org/10.2147/NDT.S19617> | Lepine, J. P., & Briley, M. (2011). The increasing burden of depression. *Neuropsychiatric Disease and Treatment, 7*(Suppl 1), 3–7. <https://doi.org/10.2147/NDT.S19617> |
| Correct | Li, M., D’Arcy, C., & Meng, X. (2016). Maltreatment in childhood substantially increases the risk of adult depression and anxiety in prospective cohort studies: Systematic review, meta-analysis, and proportional attributable fractions. *Psychological Medicine, 46*(4), 717–730. <https://doi.org/10.1017/S0033291715002743> | Li, M., D’Arcy, C., & Meng, X. (2016). Maltreatment in childhood substantially increases the risk of adult depression and anxiety in prospective cohort studies: Systematic review, meta-analysis, and proportional attributable fractions. *Psychological Medicine, 46*(4), 717–730. <https://doi.org/10.1017/S0033291715002743> |
| Fabricated | Markowitz, J. C., & Weissman, M. M. (2012). *Interpersonal psychotherapy for depression: A meta-analysis*. World Psychiatry, 11(1), 11–18. | None found |
| Correct | Miller, A. H., & Raison, C. L. (2016). The role of inflammation in depression: From evolutionary imperative to modern treatment target. *Nature Reviews Immunology, 16*(1), 22–34. <https://doi.org/10.1038/nri.2015.5> | Miller, A. H., & Raison, C. L. (2016). The role of inflammation in depression: From evolutionary imperative to modern treatment target. *Nature Reviews Immunology, 16*(1), 22–34. <https://doi.org/10.1038/nri.2015.5> |
| Correct | Moncrieff, J., Cooper, R. E., Stockmann, T., Amendola, S., Hengartner, M. P., & Horowitz, M. A. (2022). The serotonin theory of depression: A systematic umbrella review of the evidence. *Molecular Psychiatry, 27*, 2401–2413. <https://doi.org/10.1038/s41380-022-01661-0> | Moncrieff, J., Cooper, R. E., Stockmann, T., Amendola, S., Hengartner, M. P., & Horowitz, M. A. (2022). The serotonin theory of depression: A systematic umbrella review of the evidence. *Molecular Psychiatry, 27*, 2401–2413. <https://doi.org/10.1038/s41380-022-01661-0> |
| Correct | Moussavi, S., Chatterji, S., Verdes, E., Tandon, A., Patel, V., & Ustun, B. (2007). Depression, chronic diseases, and decrements in health: Results from the World Health Surveys. *The Lancet, 370*(9590), 851–858. <https://doi.org/10.1016/S0140-6736(07)61415-9> | Moussavi, S., Chatterji, S., Verdes, E., Tandon, A., Patel, V., & Ustun, B. (2007). Depression, chronic diseases, and decrements in health: Results from the World Health Surveys. *The Lancet, 370*(9590), 851–858. <https://doi.org/10.1016/S0140-6736(07)61415-9> |
| Correct | Nolen-Hoeksema, S., Wisco, B. E., & Lyubomirsky, S. (2008). Rethinking rumination. *Perspectives on Psychological Science, 3*(5), 400–424. <https://doi.org/10.1111/j.1745-6924.2008.00088.x> | Nolen-Hoeksema, S., Wisco, B. E., & Lyubomirsky, S. (2008). Rethinking rumination. *Perspectives on Psychological Science, 3*(5), 400–424. <https://doi.org/10.1111/j.1745-6924.2008.00088.x> |
| Correct | Pariante, C. M., & Lightman, S. L. (2008). The HPA axis in major depression: Classical theories and new developments. *Trends in Neurosciences, 31*(9), 464–468. <https://doi.org/10.1016/j.tins.2008.06.006> | Pariante, C. M., & Lightman, S. L. (2008). The HPA axis in major depression: Classical theories and new developments. *Trends in Neurosciences, 31*(9), 464–468. <https://doi.org/10.1016/j.tins.2008.06.006> |
| Correct | Ruhé, H. G., Mason, N. S., & Schene, A. H. (2007). Mood is indirectly related to serotonin, norepinephrine and dopamine levels in humans: A meta-analysis of monoamine depletion studies. *Molecular Psychiatry, 12*(4), 331–359. <https://doi.org/10.1038/sj.mp.4001949> | Ruhé, H. G., Mason, N. S., & Schene, A. H. (2007). Mood is indirectly related to serotonin, norepinephrine and dopamine levels in humans: A meta-analysis of monoamine depletion studies. *Molecular Psychiatry, 12*(4), 331–359. <https://doi.org/10.1038/sj.mp.4001949> |
| Non-fabricated but errors | Rude, S. S., Maestas, K. L., & Neff, K. (2007). Paying attention to distress: What’s wrong with rumination? *Cognition and Emotion, 21*(4), 843–864. [**https://doi.org/10.1080/02699930601056965**](https://doi.org/10.1080/02699930601056965) | Rude, S., Little Maestas, K., & Neff, K. (2007). Paying attention to distress: What’s wrong with rumination? *Cognition & Emotion*, *21*(4), 843–864. [**https://doi.org/10.1080/02699930601056732**](https://doi.org/10.1080/02699930601056732) |
| Correct | van Straten, A., Hill, J., Richards, D. A., & Cuijpers, P. (2015). Stepped care treatment delivery for depression: A systematic review and meta-analysis. *Psychological Medicine, 45*(2), 231–246. <https://doi.org/10.1017/S0033291714000701> | van Straten, A., Hill, J., Richards, D. A., & Cuijpers, P. (2015). Stepped care treatment delivery for depression: A systematic review and meta-analysis. *Psychological Medicine, 45*(2), 231–246. <https://doi.org/10.1017/S0033291714000701> |
|  | **SPECIALIZED REVIEW ON MAJOR DEPRESSIVE DISORDER** |  |
| Correct | Andersson, G., Cuijpers, P., Carlbring, P., Riper, H., & Hedman, E. (2014). Guided internet-based vs. face-to-face cognitive behavior therapy for psychiatric and somatic disorders: A systematic review and meta-analysis. World Psychiatry, 13(3), 288–295. <https://doi.org/10.1002/wps.20151> | Andersson, G., Cuijpers, P., Carlbring, P., Riper, H., & Hedman, E. (2014). Guided internet-based vs. face-to-face cognitive behavior therapy for psychiatric and somatic disorders: A systematic review and meta-analysis. World Psychiatry, 13(3), 288–295. <https://doi.org/10.1002/wps.20151> |
| Non-fabricated but errors | Andersson, G., Titov, N., Dear, B. F., Rozental, A., & Carlbring, P. (**2013).** Internet-delivered psychological treatments: From innovation to implementation. World Psychiatry, **12(2), 123–131.** [**https://doi.org/10.1002/wps.20092**](https://doi.org/10.1002/wps.20092) | Andersson, G., Titov, N., Dear, B. F., Rozental, A., & Carlbring, P. **(2019).** Internet‐delivered psychological treatments: From innovation to implementation. *World Psychiatry*, ***18*(1), 20–28.** [**https://doi.org/10.1002/wps.20610**](https://doi.org/10.1002/wps.20610) |
| Correct | Baumeister, H., Reichler, L., Munzinger, M., & Lin, J. (2014). The impact of guidance on Internet-based mental health interventions—A systematic review. Internet Interventions, 1(4), 205–215. <https://doi.org/10.1016/j.invent.2014.08.003> | Baumeister, H., Reichler, L., Munzinger, M., & Lin, J. (2014). The impact of guidance on Internet-based mental health interventions—A systematic review. Internet Interventions, 1(4), 205–215. <https://doi.org/10.1016/j.invent.2014.08.003> |
| Non-fabricated but errors | **Berger, T., Caspar, F., Richardson, R., Knecht, S., & Sutter, D.** (2011). Internet-based treatment of depression: A randomized controlled trial comparing guided with unguided self-help. Cognitive Behaviour Therapy, 40(4), 251–266. <https://doi.org/10.1080/16506073.2011.616531> | **Berger, T., Hämmerli, K., Gubser, N., Andersson, G., & Caspar, F.** (2011). Internet-Based Treatment of Depression: A Randomized Controlled Trial Comparing Guided with Unguided Self-Help. *Cognitive Behaviour Therapy*, *40*(4), 251–266. <https://doi.org/10.1080/16506073.2011.616531> |
| Non-fabricated but errors | **Berger, T., Krieger, T., Sude, K., Meyer, B., & Maercker, A. (2018).** Internet-based guided self-help for several anxiety disorders: A randomized controlled trial comparing a tailored with a standardized treatment. **Psychotherapy and Psychosomatics, 87(5), 296–299. https://doi.org/10.1159/000492444** | **Berger, T., Boettcher, J., & Caspar, F. (2014).** Internet-based guided self-help for several anxiety disorders: A randomized controlled trial comparing a tailored with a standardized disorder-specific approach. ***Psychotherapy*,** ***51*(2), 207–219. https://doi.org/10.1037/a0032527** |
| Correct | Buntrock, C., Ebert, D. D., Lehr, D., Smit, F., Riper, H., Berking, M., & Cuijpers, P. (2016). Effect of a web-based guided self-help intervention for prevention of major depression in adults with subthreshold depression: A randomized clinical trial. JAMA, 315(17), 1854–1863. <https://doi.org/10.1001/jama.2016.4326> | Buntrock, C., Ebert, D. D., Lehr, D., Smit, F., Riper, H., Berking, M., & Cuijpers, P. (2016). Effect of a web-based guided self-help intervention for prevention of major depression in adults with subthreshold depression: A randomized clinical trial. JAMA, 315(17), 1854–1863. <https://doi.org/10.1001/jama.2016.4326> |
| Correct | Christensen, H., Griffiths, K. M., & Farrer, L. (2009). Adherence in internet interventions for anxiety and depression: Systematic review. Journal of Medical Internet Research, 11(2), e13. <https://doi.org/10.2196/jmir.1194> | Christensen, H., Griffiths, K. M., & Farrer, L. (2009). Adherence in internet interventions for anxiety and depression: Systematic review. Journal of Medical Internet Research, 11(2), e13. <https://doi.org/10.2196/jmir.1194> |
| Non-fabricated but errors | Clarke, G., Reid, E., Eubanks, D., O'Connor, E., DeBar, L. L., Kelleher, C., Lynch, F., & Nunley, S. **(2009).** Overcoming depression on the internet (ODIN): A randomized controlled trial of an Internet depression skills intervention program. **Behaviour Research and Therapy, 47(4), 277–286.** [**https://doi.org/10.1016/j.brat.2008.12.007**](https://doi.org/10.1016/j.brat.2008.12.007) | Clarke, G., Reid, E., Eubanks, D., O’Connor, E., DeBar, L. L., Kelleher, C., Lynch, F., & Nunley, S. **(2002).** Overcoming depression on the Internet (ODIN): a randomized controlled trial of an Internet depression skills intervention program**.** ***Journal of Medical Internet Research*,** ***4*(3), E14. https://doi.org/10.2196/jmir.4.3.e14** |
| Correct | Donker, T., Petrie, K., Proudfoot, J., Clarke, J., Birch, M. R., & Christensen, H. (2013). Smartphones for smarter delivery of mental health programs: A systematic review. Journal of Medical Internet Research, 15(11), e247. <https://doi.org/10.2196/jmir.2791> | Donker, T., Petrie, K., Proudfoot, J., Clarke, J., Birch, M.-R., & Christensen, H. (2013). Smartphones for smarter delivery of mental health programs: a systematic review. Journal of Medical Internet Research, 15(11), e247. <https://doi.org/10.2196/jmir.2791> |
| Correct | Firth, J., Torous, J., Nicholas, J., Carney, R., Rosenbaum, S., & Sarris, J. (2017). Can smartphone mental health interventions reduce symptoms of anxiety? A meta-analysis of randomized controlled trials. Journal of Affective Disorders, 218, 15–22. <https://doi.org/10.1016/j.jad.2017.04.046> | Firth, J., Torous, J., Nicholas, J., Carney, R., Rosenbaum, S., & Sarris, J. (2017). Can smartphone mental health interventions reduce symptoms of anxiety? A meta-analysis of randomized controlled trials. Journal of Affective Disorders, 218, 15–22. <https://doi.org/10.1016/j.jad.2017.04.046> |
| Non-fabricated but errors | Furukawa, T. A., Noma, H., Caldwell, D. M., Honyashiki, M., Shinohara, K., Imai, H., Chen, P., Hunot, V., & Churchill, R. **(2018). Waiting list may substantially overestimate the effect size of cognitive-behavioral therapy for depression: A meta-analysis. Scientific Reports, 6, 30199.** [**https://doi.org/10.1038/srep30199**](https://doi.org/10.1038/srep30199) | Furukawa, T. A., Noma, H., Caldwell, D. M., Honyashiki, M., Shinohara, K., Imai, H., Chen, P., Hunot, V., & Churchill, R. **(2014).** **Waiting list may be a nocebo condition in psychotherapy trials: a contribution from network meta-analysis**. ***Acta Psychiatrica Scandinavica*,** ***130*(3), 181–192. https://doi.org/10.1111/acps.12275** |
| Non-fabricated but errors | Griffiths, K. M., Farrer, L., & Christensen, H. **(2012).** The efficacy of internet interventions for depression and anxiety disorders: A review of randomised controlled trials. Medical Journal of Australia, 192(11 Suppl), S4–S11. <https://doi.org/10.5694/j.1326-5377.2010.tb03685.x> | Griffiths, K. M., Farrer, L., & Christensen, H. **(2010).** The efficacy of internet interventions for depression and anxiety disorders: a review of randomised controlled trials. *The Medical Journal of Australia*, *192*(S11), S4–S11. <https://doi.org/10.5694/j.1326-5377.2010.tb03685.x> |
| Fabricated | Jacobson, N. C., Weingarden, H., & Wilhelm, S. (2020). Digital psychotherapies in the COVID-19 era: A call to action. Nature Partner Journals: Digital Medicine, 3, 90. <https://doi.org/10.1038/s41746-020-0305-1> | None found |
| Correct | Kazdin, A. E., & Rabbitt, S. M. (2013). Novel models for delivering mental health services and reducing the burdens of mental illness. Clinical Psychological Science, 1(2), 170–191. <https://doi.org/10.1177/2167702612463566> | Kazdin, A. E., & Rabbitt, S. M. (2013). Novel models for delivering mental health services and reducing the burdens of mental illness. Clinical Psychological Science, 1(2), 170–191. <https://doi.org/10.1177/2167702612463566> |
| Non-fabricated but errors | Karyotaki, E., Efthimiou, O., Miguel, C., Bermpohl, F. M. G., Furukawa, T. A., Cuijpers, P., (2021). Internet-based cognitive behavioral therapy for depression: A systematic review and individual participant data network meta-analysis. JAMA Psychiatry, 78(4), 361–371. [**https://doi.org/10.1001/jamapsychiatry.2020.4368**](https://doi.org/10.1001/jamapsychiatry.2020.4368) | Karyotaki, E., Efthimiou, O., Miguel, C., Bermpohl, F. M. G., Furukawa, T. A., Cuijpers, P., Riper, H., Patel, V., Mira, A., Gemmil, A. W., Yeung, A. S., Lange, A., Williams, A. D., Mackinnon, A., Geraedts, A., van Straten, A., Meyer, B., Björkelund, C., Knaevelsrud, C., … Forsell, Y. (2021). Internet-Based Cognitive Behavioral Therapy for Depression: A Systematic Review and Individual Patient Data Network Meta-analysis. *JAMA Psychiatry*, *78*(4), 361–371. **https://doi.org/10.1001/jamapsychiatry.2020.4364** |
| Correct | Karyotaki, E., Riper, H., Twisk, J., Hoogendoorn, A., Kleiboer, A., Mira, A., MacKinnon, D., Meyer, B., Botella, C., Littlewood, E., Andersson, G., Christensen, H., Klein, J. P., Schröder, J., Bretón-López, J., Scheider, J., Griffiths, K., Farrer, L., Huibers, M. J. H., ... Cuijpers, P. (2017). Efficacy of self-guided internet-based cognitive behavioral therapy in the treatment of depressive symptoms: A meta-analysis of individual participant data. JAMA Psychiatry, 74(4), 351–359. <https://doi.org/10.1001/jamapsychiatry.2017.0044> | Karyotaki, E., Riper, H., Twisk, J., Hoogendoorn, A., Kleiboer, A., Mira, A., MacKinnon, D., Meyer, B., Botella, C., Littlewood, E., Andersson, G., Christensen, H., Klein, J. P., Schröder, J., Bretón-López, J., Scheider, J., Griffiths, K., Farrer, L., Huibers, M. J. H., ... Cuijpers, P. (2017). Efficacy of self-guided internet-based cognitive behavioral therapy in the treatment of depressive symptoms: A meta-analysis of individual participant data. JAMA Psychiatry, 74(4), 351–359. <https://doi.org/10.1001/jamapsychiatry.2017.0044> |
| Correct | Klein, J. P., Berger, T., Schröder, J., Späth, C., Meyer, B., Caspar, F., Hautzinger, M., & Lutz, W et al. (2016). Effects of a psychological internet intervention in the treatment of mild to moderate depressive symptoms: Results of the EVIDENT study, a randomized controlled trial. Psychotherapy and Psychosomatics, 85(4), 218–228. <https://doi.org/10.1159/000445355> | Klein, J. P., Berger, T., Schröder, J., Späth, C., Meyer, B., Caspar, F., Hautzinger, M., & Lutz, W et al. (2016). Effects of a psychological internet intervention in the treatment of mild to moderate depressive symptoms: Results of the EVIDENT study, a randomized controlled trial. Psychotherapy and Psychosomatics, 85(4), 218–228. <https://doi.org/10.1159/000445355> |
| Non-fabricated but errors | Kuyken, W., Warren, F. C., Taylor, R. S., Whalley, B., Crane, C., Bondolfi, G., Hayes, R., Huijbers, M., Ma, H., Schweizer, S., Segal, Z., Speckens, A., & Teasdale, J. D. et al (2016). Efficacy of mindfulness-based cognitive therapy in prevention of depressive relapse: An individual patient data meta-analysis from randomized trials. **The Lancet, 386(9988), 63–73.** [**https://doi.org/10.1016/S0140-6736(14)62222-3**](https://doi.org/10.1016/S0140-6736(14)62222-3) | Kuyken, W., Warren, F. C., Taylor, R. S., Whalley, B., Crane, C., Bondolfi, G., Hayes, R., Huijbers, M., Ma, H., Schweizer, S., Segal, Z., Speckens, A., Teasdale, J. D., Van Heeringen, K., Williams, M., Byford, S., Byng, R., & Dalgleish, T. (2016). Efficacy of Mindfulness-Based Cognitive Therapy in Prevention of Depressive Relapse: An Individual Patient Data Meta-analysis From Randomized Trials. ***JAMA Psychiatry*,** ***73*(6), 565–574. https://doi.org/10.1001/jamapsychiatry.2016.0076** |
| Non-fabricated but errors | **Lecomte, T., Potvin, S., Khazaal, Y., Chatard, A., & Orri, M. (**2020). Mobile apps for mental health issues: Meta-review of meta-analyses. JMIR mHealth and uHealth, 8(5), e17458. <https://doi.org/10.2196/17458> | **Lecomte, T., Potvin, S., Corbière, M., Guay, S., Samson, C., Cloutier, B., Francoeur, A., Pennou, A., & Khazaal, Y.** (2020). Mobile Apps for Mental Health Issues: Meta-Review of Meta-Analyses. *JMIR MHealth and UHealth*, *8*(5), e17458. <https://doi.org/10.2196/17458> |
| Fabricated | Leigh, S., & Flatt, S. (2015). App-based interventions for anxiety and depression: Systematic review and meta-analysis. Journal of Depression and Anxiety, 4(4), 2167–1044. <https://doi.org/10.4172/2167-1044.1000195> | None found |
| Correct | Linardon, J., Cuijpers, P., Carlbring, P., Messer, M., & Fuller-Tyszkiewicz, M. (2019). The efficacy of app-supported smartphone interventions for mental health problems: A meta-analysis of randomized controlled trials. World Psychiatry, 18(3), 325–336. <https://doi.org/10.1002/wps.20673> | Linardon, J., Cuijpers, P., Carlbring, P., Messer, M., & Fuller-Tyszkiewicz, M. (2019). The efficacy of app-supported smartphone interventions for mental health problems: A meta-analysis of randomized controlled trials. World Psychiatry, 18(3), 325–336. <https://doi.org/10.1002/wps.20673> |
| Non-fabricated but errors | Linardon, J., & Fuller-Tyszkiewicz, M. (2020). **Attrition and adherence in smartphone-delivered interventions for mental health problems: A meta-analysis**. **Psychological Assessment, 32(12), 1192–1202.** [**https://doi.org/10.1037/pas0000820**](https://doi.org/10.1037/pas0000820) | Linardon, J., & Fuller-Tyszkiewicz, M. (2020). **Attrition and adherence in smartphone-delivered interventions for mental health problems: A systematic and meta-analytic review**. ***Journal of Consulting and Clinical Psychology*,** ***88*(1), 1–13. https://doi.org/10.1037/ccp0000459** |
| Correct | Melville, K. M., Casey, L. M., & Kavanagh, D. J. (2010). Dropout from Internet-based treatment for psychological disorders. British Journal of Clinical Psychology, 49(4), 455–471. <https://doi.org/10.1348/014466509X472138> | Melville, K. M., Casey, L. M., & Kavanagh, D. J. (2010). Dropout from Internet-based treatment for psychological disorders. British Journal of Clinical Psychology, 49(4), 455–471. <https://doi.org/10.1348/014466509X472138> |
| Correct | Mohr, D. C., Cuijpers, P., & Lehman, K. (2011). Supportive accountability: A model for providing human support to enhance adherence to eHealth interventions. Journal of Medical Internet Research, 13(1), e30. <https://doi.org/10.2196/jmir.1602> | Mohr, D. C., Cuijpers, P., & Lehman, K. (2011). Supportive accountability: a model for providing human support to enhance adherence to eHealth interventions. Journal of Medical Internet Research, 13(1), e30. https://doi.org/10.2196/jmir.1602 |
| Correct | Nahum-Shani, I., Smith, S. N., Spring, B. J., Collins, L. M., Witkiewitz, K., Tewari, A., & Murphy, S. A. (2018). Just-in-time adaptive interventions (JITAIs) in mobile health: Key components and design principles for ongoing health behavior support. Annals of Behavioral Medicine, 52(6), 446–462. https://doi.org/10.1007/s12160-016-9830-8 | Nahum-Shani, I., Smith, S. N., Spring, B. J., Collins, L. M., Witkiewitz, K., Tewari, A., & Murphy, S. A. (2018). Just-in-time adaptive interventions (JITAIs) in mobile health: Key components and design principles for ongoing health behavior support. Annals of Behavioral Medicine, 52(6), 446–462. https://doi.org/10.1007/s12160-016-9830-8 |
| Non-fabricated but errors | Perski, O., Blandford, A., West, R., & Michie, S. (2017). Conceptualising engagement with digital behaviour change interventions: A systematic review using principles from critical interpretive synthesis. **Digital Health, 3, 2055207616671375.** [**https://doi.org/10.1177/2055207616671375**](https://doi.org/10.1177/2055207616671375) | Perski, O., Blandford, A., West, R., & Michie, S. (2017). Conceptualising engagement with digital behaviour change interventions: A systematic review using principles from critical interpretive synthesis. ***Translational Behavioral Medicine*,** ***7*(2), 254–267. https://doi.org/10.1007/s13142-016-0453-1** |
| Correct | Richards, D., & Richardson, T. (2012). Computer-based psychological treatments for depression: A systematic review and meta-analysis. Clinical Psychology Review, 32(4), 329–342. <https://doi.org/10.1016/j.cpr.2012.02.004> | Richards, D., & Richardson, T. (2012). Computer-based psychological treatments for depression: A systematic review and meta-analysis. Clinical Psychology Review, 32(4), 329–342. <https://doi.org/10.1016/j.cpr.2012.02.004> |
| Fabricated | Schleider, J. L., Abel, M. R., & Weisz, J. R. (2022). Single-session interventions for mental health: Current status and future directions. Annual Review of Clinical Psychology, 18, 351–378. <https://doi.org/10.1146/annurev-clinpsy-081219-102012> | None found |
| Non-fabricated but errors | Torous, J., Lipschitz, J., Ng, M., & Firth, J. (2020). Dropout rates in clinical trials of smartphone apps for depressive symptoms: A systematic review and meta-analysis. Journal of Affective Disorders, 263, 413–419. [**https://doi.org/10.1016/j.jad.2019.11.067**](https://doi.org/10.1016/j.jad.2019.11.067) | Torous, J., Lipschitz, J., Ng, M., & Firth, J. (2020). Dropout rates in clinical trials of smartphone apps for depressive symptoms: A systematic review and meta-analysis. *Journal of Affective Disorders*, *263*, 413–419. **https://doi.org/10.1016/j.jad.2019.11.167** |
| Non-fabricated but errors | **Torous, J., Wisniewski, H., Bird, B., Carpenter, E., David, G., Elejalde, E., Firth, J., & Haim, A. (2018).** **Creating a digital health smartphone app and digital phenotyping platform for mental health and diverse healthcare needs: An interdisciplinary approach**. Journal of Technology in Behavioral Science**, 3(**2), 73–85. [**https://doi.org/10.1007/s41347-018-0040-1**](https://doi.org/10.1007/s41347-018-0040-1) | **Torous, J., Wisniewski, H., Bird, B., Carpenter, E., David, G., Elejalde, E., Fulford, D., Guimond, S., Hays, R., Henson, P., Hoffman, L., Lim, C., Menon, M., Noel, V., Pearson, J., Peterson, R., Susheela, A., Troy, H., Vaidyam, A., … Keshavan, M. (2019**). **Creating a Digital Health Smartphone App and Digital Phenotyping Platform for Mental Health and Diverse Healthcare Needs: an Interdisciplinary and Collaborative Approach**. *Journal of Technology in Behavioral Science*, ***4***(2), 73–85. **https://doi.org/10.1007/s41347-019-00095-w** |
| Correct | Vos, T., Abajobir, A. A., Abate, K. H., Abbafati, C., Abbas, K. M., Abd-Allah, F., Abera, S. F., Aboyans, V., Adetokunboh, O., Afshin, A., Agrawal, A., Kassebaum, N. J., et al. (2017). Global, regional, and national incidence, prevalence, and years lived with disability for 328 diseases and injuries for 195 countries: A systematic analysis for the Global Burden of Disease Study 2016. The Lancet, 390(10100), 1211–1259. <https://doi.org/10.1016/S0140-6736(17)32154-2> | Vos, T., Abajobir, A. A., Abate, K. H., Abbafati, C., Abbas, K. M., Abd-Allah, F., Abera, S. F., Aboyans, V., Adetokunboh, O., Afshin, A., Agrawal, A., Kassebaum, N. J., et al. (2017). Global, regional, and national incidence, prevalence, and years lived with disability for 328 diseases and injuries for 195 countries: A systematic analysis for the Global Burden of Disease Study 2016. The Lancet, 390(10100), 1211–1259. <https://doi.org/10.1016/S0140-6736(17)32154-2> |
| Non-fabricated but errors | **Weisel, K. K., Fuhrmann, L. M., Berking, M., Baumeister, H., Cuijpers, P., Ebert, D. D., & Riper, H**. (2019). Standalone smartphone apps for mental health: A systematic review and meta-analysis. **World Psychiatry, 18(3), 301–311. https://doi.org/10.1002/wps.20650** | **Weisel, K. K., Fuhrmann, L. M., Berking, M., Baumeister, H., Cuijpers, P., & Ebert, D. D.** (2019). Standalone smartphone apps for mental health-a systematic review and meta-analysis. ***NPJ Digital Medicine*,** ***2*, 118. https://doi.org/10.1038/s41746-019-0188-8** |
| Non-fabricated but errors | Yardley, L., Morrison, L., Bradbury, K., & Muller, I. **(2016).** The person-based approach to intervention development: Application to digital health-related behavior change interventions. Journal of Medical Internet Research, 18(1), e30. <https://doi.org/10.2196/jmir.4055> | Yardley, L., Morrison, L., Bradbury, K., & Muller, I. **(2015).** The person-based approach to intervention development: application to digital health-related behavior change interventions. *Journal of Medical Internet Research*, ***17***(1), e30. https://doi.org/10.2196/jmir.4055 |
|  | **SPECIALIZED REVIEW ON BODY DYSMORPHIC DISORDER** |  |
| Correct | Andersson, G., Carlbring, P., Titov, N., & Lindefors, N. (2019). Internet interventions for adults with anxiety and mood disorders: A narrative umbrella review of recent meta-analyses. The Canadian Journal of Psychiatry, 64(7), 465–470. <https://doi.org/10.1177/0706743719839381> | Andersson, G., Carlbring, P., Titov, N., & Lindefors, N. (2019). Internet interventions for adults with anxiety and mood disorders: A narrative umbrella review of recent meta-analyses. The Canadian Journal of Psychiatry, 64(7), 465–470. <https://doi.org/10.1177/0706743719839381> |
| Correct | American Psychiatric Association. (2013). *Diagnostic and statistical manual of mental disorders* (5th ed.). <https://doi.org/10.1176/appi.books.9780890425596> | American Psychiatric Association. (2013). *Diagnostic and statistical manual of mental disorders* (5th ed.). <https://doi.org/10.1176/appi.books.9780890425596> |
| Correct | Carlbring, P., Andersson, G., Cuijpers, P., Riper, H., & Hedman-Lagerlöf, E. (2018). Internet-based vs. face-to-face cognitive behavior therapy for psychiatric and somatic disorders: An updated systematic review and meta-analysis. *Cognitive Behaviour Therapy, 47*(1), 1–18. <https://doi.org/10.1080/16506073.2017.1401115> | Carlbring, P., Andersson, G., Cuijpers, P., Riper, H., & Hedman-Lagerlöf, E. (2018). Internet-based vs. face-to-face cognitive behavior therapy for psychiatric and somatic disorders: An updated systematic review and meta-analysis. *Cognitive Behaviour Therapy, 47*(1), 1–18. <https://doi.org/10.1080/16506073.2017.1401115> |
| Non-fabricated but errors | **Enander, J., Ivanov, V. Z., Mataix-Cols, D., Kuja-Halkola, R., Ljótsson, B., Rück, C., & Andersson, E. (2019).** **Outcome predictors in guided internet-based cognitive–behavioral therapy for body dysmorphic disorder: A machine learning approach**. *BMC Psychiatry,* ***19*, 433.** [**https://doi.org/10.1186/s12888-019-2409-0**](https://doi.org/10.1186/s12888-019-2409-0) | **Flygare, O., Enander, J., Andersson, E., Ljótsson, B., Ivanov, V. Z., Mataix-Cols, D., & Rück, C**. **(2020).** **Predictors of remission from body dysmorphic disorder after internet-delivered cognitive behavior therapy: a machine learning approach.** *BMC Psychiatry*, ***20*(1), 1–9. https://doi.org/10.1186/s12888-020-02655-4** |
| Non-fabricated but errors | **Enander, J., Ljótsson, B., Andersson, E., Rück, C., Lindefors, N., Andersson, G., ... & Mataix-Cols, D.** (2016). **Therapist-guided, internet-based cognitive–behavioral therapy for body dysmorphic disorder: A randomized controlled trial.** ***The American Journal of Psychiatry, 173*(7), 647–656.** [**https://doi.org/10.1176/appi.ajp.2015.15081061**](https://doi.org/10.1176/appi.ajp.2015.15081061) | **Enander, J., Andersson, E., Mataix-Cols, D., Lichtenstein, L., Alström, K., Andersson, G., Ljótsson, B., & Rück, C.** (2016). **Therapist guided internet based cognitive behavioural therapy for body dysmorphic disorder: single blind randomised controlled trial.** ***BMJ (Clinical Research Ed.)*,** ***352*, i241. https://doi.org/10.1136/bmj.i241** |
| Correct | Linardon, J., & Fuller-Tyszkiewicz, M. (2020). Attrition and adherence in smartphone-delivered interventions for mental health problems: A systematic and meta-analytic review. *Journal of Consulting and Clinical Psychology, 88*(1), 1–13. <https://doi.org/10.1037/ccp0000459> | Linardon, J., & Fuller-Tyszkiewicz, M. (2020). Attrition and adherence in smartphone-delivered interventions for mental health problems: A systematic and meta-analytic review. *Journal of Consulting and Clinical Psychology, 88*(1), 1–13. <https://doi.org/10.1037/ccp0000459> |
| Fabricated | Linardon, J., Shatte, A., Tepper, H., & Fuller-Tyszkiewicz, M. (2019). A systematic evaluation of popular smartphone apps for the treatment of mental health problems: Are apps evidence-based? *Behavior Research and Therapy, 111*, 1–9. <https://doi.org/10.1016/j.brat.2018.10.012> | None found |
| Non-fabricated but errors | **Lindner, P., Miloff, A., Hamilton, W., Reuterskiöld, L., Andersson, G., & Carlbring, P. (2020).** Creating state-of-the-art, next-generation virtual reality exposure therapies for anxiety disorders using consumer hardware platforms: Design considerations and future directions. *Cognitive Behaviour Therapy,* ***49***(5), **365–380.** [**https://doi.org/10.1080/16506073.2020.1807933**](https://doi.org/10.1080/16506073.2020.1807933) | **Lindner, P., Miloff, A., Hamilton, W., Reuterskiöld, L., Andersson, G., Powers, M. B., & Carlbring, P. (2017).** Creating state of the art, next-generation Virtual Reality exposure therapies for anxiety disorders using consumer hardware platforms: design considerations and future directions. *Cognitive Behaviour Therapy*, ***46*(**5), **404–420.** [**https://doi.org/10.1080/16506073.2017.1280843**](https://doi.org/10.1080/16506073.2017.1280843) |
| Correct | Olthuis, J. V., Watt, M. C., Bailey, K., Hayden, J. A., & Stewart, S. H. (2016). Therapist-supported Internet cognitive behavioural therapy for anxiety disorders in adults. *Cochrane Database of Systematic Reviews, 3*, CD011565. <https://doi.org/10.1002/14651858.CD011565.pub2> | Olthuis, J. V., Watt, M. C., Bailey, K., Hayden, J. A., & Stewart, S. H. (2016). Therapist-supported Internet cognitive behavioural therapy for anxiety disorders in adults. *Cochrane Database of Systematic Reviews, 3*, CD011565. <https://doi.org/10.1002/14651858.CD011565.pub2> |
| Fabricated | Rück, C., Enander, J., Jangmo, A., Hedman-Lagerlöf, E., Ljótsson, B., Lindefors, N., ... & Mataix-Cols, D. (2019). Effect of internet-based cognitive behavior therapy vs face-to-face cognitive behavior therapy on symptom severity in body dysmorphic disorder: A randomized clinical trial. *JAMA Psychiatry, 76*(10), 987–995. <https://doi.org/10.1001/jamapsychiatry.2019.2159> | None found |
| Fabricated | Schneider, S. C., Von Brachel, R., Hohwy, J., & Zimmermann, P. (2022). Mobile self-help for appearance-related self-criticism: A randomized controlled pilot trial. *Internet Interventions, 29*, 100555. <https://doi.org/10.1016/j.invent.2022.100555> | None found |
| Non-fabricated but errors | Schulte, F. P., Schulze, L., Buhlmann, U., & Kathmann, N. (2020). **Body dysmorphic disorder: Treatment barriers, motivation, and the need for a stepped-care approach**. ***Frontiers in Psychiatry, 11*, 570249.** [**https://doi.org/10.3389/fpsyt.2020.570249**](https://doi.org/10.3389/fpsyt.2020.570249) | Schulte, J., Schulz, C., Wilhelm, S., & Buhlmann, U. (2020). **Treatment utilization and treatment barriers in individuals with body dysmorphic disorder**. ***BMC Psychiatry*,** ***20*(1), 1–11. https://doi.org/10.1186/s12888-020-02489-0** |
| Correct | Torous, J., Lipschitz, J., Ng, M., & Firth, J. (2020). Dropout rates in clinical trials of smartphone apps for depressive symptoms: A systematic review and meta-analysis. *Journal of Affective Disorders, 263*, 413–419. <https://doi.org/10.1016/j.jad.2019.11.167> | Torous, J., Lipschitz, J., Ng, M., & Firth, J. (2020). Dropout rates in clinical trials of smartphone apps for depressive symptoms: A systematic review and meta-analysis. *Journal of Affective Disorders, 263*, 413–419. <https://doi.org/10.1016/j.jad.2019.11.167> |
| Correct | Veale, D., Gledhill, L. J., Christodoulou, P., & Hodsoll, J. (2016). Body dysmorphic disorder in different settings: A systematic review and estimated weighted prevalence. *Body Image, 18*, 168–186. <https://doi.org/10.1016/j.bodyim.2016.07.003> | Veale, D., Gledhill, L. J., Christodoulou, P., & Hodsoll, J. (2016). Body dysmorphic disorder in different settings: A systematic review and estimated weighted prevalence. *Body Image, 18*, 168–186. <https://doi.org/10.1016/j.bodyim.2016.07.003> |
| Non-fabricated but errors | **Veale, D., Neziroglu, F., & Phillips, K. A. (2021**). *Body dysmorphic disorder: Advances in research and clinical practice*. Oxford University Press. [**https://doi.org/10.1093/med-psych/9780198810980.001.0001**](https://doi.org/10.1093/med-psych/9780198810980.001.0001) | **Phillips, K. A. (Ed.). (2017).** *Body dysmorphic disorder: Advances in research and clinical practice*. Oxford University Press. [**https://doi.org/10.1093/med/9780190254131.001.0001**](https://doi.org/10.1093/med/9780190254131.001.0001) |
| Fabricated | Weingarden, H., Tangney, J. P., & Wilhelm, S. (2021). Evaluation of a mobile app for body image and body dysmorphic symptoms: A randomized pilot trial. *Journal of Obsessive-Compulsive and Related Disorders, 28*, 100615. <https://doi.org/10.1016/j.jocrd.2020.100615> | None found |
| Non-fabricated but errors | **Wilhelm, S., Phillips, K. A., Fama, J. M., Greenberg, J. L., & Steketee, G. (**2014). Modular cognitive-behavioral therapy for body dysmorphic disorder: A randomized controlled trial. *Behavior Therapy, 45*(3), 314–327. [**https://doi.org/10.1016/j.beth.2013.12.011**](https://doi.org/10.1016/j.beth.2013.12.011) | **Wilhelm, S., Phillips, K. A., Didie, E., Buhlmann, U., Greenberg, J. L., Fama, J. M., Keshaviah, A., & Steketee,** G. (2014). Modular cognitive-behavioral therapy for body dysmorphic disorder: A randomized controlled trial. *Behavior Therapy*, *45*(3), 314–327. **https://doi.org/10.1016/j.beth.2013.12.007** |
|  | **GENERAL REVIEW ON BODY DYSMORPHIC DISORDER** |  |
| Correct | American Psychiatric Association. (2013). Diagnostic and statistical manual of mental disorders (5th ed.). <https://doi.org/10.1176/appi.books.9780890425596> | American Psychiatric Association. (2013). Diagnostic and statistical manual of mental disorders (5th ed.). <https://doi.org/10.1176/appi.books.9780890425596> |
| Correct | Angelakis, I., Gooding, P., & Panagioti, M. (2016). Suicidality in body dysmorphic disorder (BDD): A systematic review with meta-analysis. Clinical Psychology Review, 49, 55–66. <https://doi.org/10.1016/j.cpr.2016.08.002> | Angelakis, I., Gooding, P., & Panagioti, M. (2016). Suicidality in body dysmorphic disorder (BDD): A systematic review with meta-analysis. Clinical Psychology Review, 49, 55–66. <https://doi.org/10.1016/j.cpr.2016.08.002> |
| Fabricated | Bartsch, M., Lanius, D., & Winter, D. (2016). Predicting body dysmorphic symptoms: The role of childhood abuse and attachment. Psychopathology, 49(4), 270–276. <https://doi.org/10.1159/000447385> | None found |
| Fabricated | Bjornsson, A. S., Didie, E. R., Phillips, K. A., & Fay, C. (2013). Body dysmorphic disorder. In J. M. Oldham, A. E. Skodol, & D. S. Bender (Eds.), The American Psychiatric Publishing textbook of personality disorders (2nd ed., pp. 497–511). American Psychiatric Publishing. | None found |
| Non-fabricated but errors | Buchanan, E., Rossell, S., & Castle, D. **(2014).** Body dysmorphic disorder: A review of nosology, cognition and neurobiology. Neuropsychiatry, **4**(1), **15–27.** [**https://doi.org/10.2217/npy.13.84**](https://doi.org/10.2217/npy.13.84) | Buchanan, B. G., Rossell, S. L., & Castle, D. J. **(2011).** Body dysmorphic disorder: a review of nosology, cognition and neurobiology.*Neuropsychiatry,****1***(1), **71-80.** https://doi.org/10.2217/npy.10.3 |
| Non-fabricated but errors | Buhlmann, U., Teachman, B. A., Gerbershagen, A., Kikul, J., & Rief, W. (2008). **Implicit and explicit self-esteem in body dysmorphic disorder.** **Journal of Behavior Therapy and Experimental Psychiatry, 39(3), 317–328.** [**https://doi.org/10.1016/j.jbtep.2007.10.001**](https://doi.org/10.1016/j.jbtep.2007.10.001) | Buhlmann, U., Teachman, B. A., Gerbershagen, A., Kikul, J., & Rief, W. (2008). **Implicit and Explicit Self-Esteem and Attractiveness Beliefs among Individuals with Body Dysmorphic Disorder.** ***Cognitive Therapy & Research*,** ***32*(2), 213–225. https://doi.org/10.1007/s10608-006-9095-9** |
| Non-fabricated but errors | Clerkin, E. M., & Teachman, B. A. **(2009).** Training implicit social anxiety associations: An experimental intervention. Journal of Anxiety Disorders, **23**(3), 300–308. [**https://doi.org/10.1016/j.janxdis.2008.09.005**](https://doi.org/10.1016/j.janxdis.2008.09.005) | Clerkin, E. M., & Teachman, B. A**. (2010).** Training implicit social anxiety associations: An experimental intervention. *Journal of Anxiety Disorders*, ***24***(3), 300–308. **https://doi.org/10.1016/j.janxdis.2010.01.001** |
| Non-fabricated but errors | Crerand, C. E., Franklin, M. E., & Sarwer, D. B**. (2005).** Body dysmorphic disorder and cosmetic surgery. Plastic and Reconstructive Surgery, **116(4), 1230–1236.** [**https://doi.org/10.1097/01.prs.0000175194.15645.98**](https://doi.org/10.1097/01.prs.0000175194.15645.98) | Crerand, C. E. , Franklin, M. E. & Sarwer, D. B.  **(2006).**  Body Dysmorphic Disorder and Cosmetic Surgery.  *Plastic and Reconstructive Surgery,****118*** **(7),** **167e-180e.** **10.1097/01.prs.0000242500.28431.24.** |
| Fabricated | Didie, E. R., Kelly, M. M., & Phillips, K. A. (2008). Family functioning in individuals with body dysmorphic disorder. Journal of Anxiety Disorders, 22(3), 549–559. <https://doi.org/10.1016/j.janxdis.2007.05.005> | Not found |
| Non-fabricated but errors | Enander, J., Ivanov, V. Z., Andersson, E., Mataix-Cols, D., Ljótsson, B., & Rück, C. **(2016).** **Therapist-guided, internet-based cognitive-behavioral therapy for body dysmorphic disorder: A randomized controlled trial**. BMJ Open, **6(4), e008341.** [**https://doi.org/10.1136/bmjopen-2015-008341**](https://doi.org/10.1136/bmjopen-2015-008341) | Enander, J., Ivanov, V. Z., Andersson, E., Mataix-Cols, D., Ljótsson, B., & Rück, C. **(2014).** **Therapist-guided, Internet-based cognitive-behavioural therapy for body dysmorphic disorder (BDD-NET): a feasibility study**. *BMJ Open*, ***4*(9), e005923. https://doi.org/10.1136/bmjopen-2014-005923** |
| Non-fabricated but errors | Fardouly, J., & Vartanian, L. R. (2016). **Social media and body image concerns.** Current Opinion in Psychology, 9, 1–5. <https://doi.org/10.1016/j.copsyc.2015.09.005> | Fardouly, J., & Vartanian, L. R. (2016). **Social media and body image concerns: Current research and future directions.** *Current Opinion in Psychology*, *9*, 1–5. https://doi.org/10.1016/j.copsyc.2015.09.005 |
| Correct | Feusner, J. D., Townsend, J., Bystritsky, A., & Bookheimer, S. (2007). Visual information processing of faces in body dysmorphic disorder. Archives of General Psychiatry, 64(12), 1417–1425. <https://doi.org/10.1001/archpsyc.64.12.1417> | Feusner, J. D., Townsend, J., Bystritsky, A., & Bookheimer, S. (2007). Visual information processing of faces in body dysmorphic disorder. Archives of General Psychiatry, 64(12), 1417–1425. <https://doi.org/10.1001/archpsyc.64.12.1417> |
| Fabricated | Hartmann, A. S., Thomas, J. J., Greenberg, J. L., Wilhelm, S., & Storch, E. A. (2015). How much is too much? The economic burden of BDD. Journal of Psychiatric Research, 68, 270–277. <https://doi.org/10.1016/j.jpsychires.2015.07.007> | Not found |
| Fabricated | Hong, K., Nezgovorova, V., & Feusner, J. D. (2018). Gender differences in BDD: A review. Clinical Psychology Review, 66, 1–12. <https://doi.org/10.1016/j.cpr.2018.09.002> | Not found |
| Non-fabricated but errors | **Hrabosky, J. I., Veale, D., & Neziroglu, F. (2009).** Body dysmorphic disorder: A treatment manual. Oxford University Press. | **Veale, D., & Neziroglu, F. (2010).** *Body dysmorphic disorder: A treatment manual*. John Wiley & Sons Ltd. https://doi.org/10.1002/9780470684610 |
| Non-fabricated but errors | Monzani, B., Rijsdijk, F., Harris, J., & Mataix-Cols, D. (2014). **The structure of genetic and environmental risk factors for body dysmorphic symptoms and obsessive–compulsive symptoms. Journal of Abnormal Psychology, 123(3), 564–574.** [**https://doi.org/10.1037/a0036776**](https://doi.org/10.1037/a0036776) | Monzani, B., Rijsdijk, F., Harris, J., & Mataix-Cols, D. (2014). **The structure of genetic and environmental risk factors for dimensional representations of DSM-5 obsessive-compulsive spectrum disorders.** ***JAMA Psychiatry*,** ***71*(2), 182–189. https://doi.org/10.1001/jamapsychiatry.2013.3524** |
| Non-fabricated but errors | **Moody, T. D., Sasaki, M., Bohon, C., et al. (**2015). Functional connectivity for face processing in individuals with body dysmorphic disorder and anorexia nervosa. Psychological Medicine, 45(16), 3491–3503. [**https://doi.org/10.1017/S003329171500138X**](https://doi.org/10.1017/S003329171500138X) | **Moody, T. D., Sasaki, M. A., Bohon, C., Strober, M. A., Bookheimer, S. Y., Sheen, C. L., & Feusner, J. D**. (2015). Functional connectivity for face processing in individuals with body dysmorphic disorder and anorexia nervosa. *Psychological Medicine*, *45*(16), 3491–3503. **https://doi.org/10.1017/S0033291715001397** |
| Non-fabricated but errors | Murray, S. B., Griffiths, S., & Mond, J. M. **(2017).** Evolving eating disorder psychopathology: Conceptualising muscularity-oriented disordered eating. British Journal of Psychiatry, **211(6), 377–379.** [**https://doi.org/10.1192/bjp.bp.116.187450**](https://doi.org/10.1192/bjp.bp.116.187450) | Murray, S. B., Griffiths, S., & Mond, J. M. **(2016).** Evolving eating disorder psychopathology: conceptualising muscularity-oriented disordered eating. *The British Journal of Psychiatry : The Journal of Mental Science*, ***208*(5), 414–415. https://doi.org/10.1192/bjp.bp.115.168427** |
| Non-fabricated but errors | Neziroglu, F., Khemlani-Patel, S., & Veale, D. **(2004).** Social learning theory and cognitive behavioral models of body dysmorphic disorder. Body Image, **1**(1), **63–69.** [**https://doi.org/10.1016/S1740-1445(03)00005-2**](https://doi.org/10.1016/S1740-1445(03)00005-2) | Neziroglu, F., Khemlani-Patel, S., & Veale, D. **(2008).** Social learning theory and cognitive behavioral models of body dysmorphic disorder. *Body Image*, ***5***(1), **28–38.** **https://doi.org/10.1016/j.bodyim.2008.01.002** |
| Non-fabricated but errors | Phillips, K. A., Menard, W., Fay, C., & Pagano, M. E. **(2006).** Psychosocial functioning and quality of life in body dysmorphic disorder. Comprehensive Psychiatry, 46(4), 254–260. [**https://doi.org/10.1016/j.comppsych.2005.09.005**](https://doi.org/10.1016/j.comppsych.2005.09.005) | Phillips, K. A., Menard, W., Fay, C., & Pagano, M. E. **(2005).** Psychosocial functioning and quality of life in body dysmorphic disorder. *Comprehensive Psychiatry*, *46*(4), 254–260. **https://doi.org/10.1016/j.comppsych.2004.10.004** |
| Non-fabricated but errors | Phillips, K. A., & Hollander, E. (2008). Treating body dysmorphic disorder with medication: Evidence and recommendations. **CNS Drugs, 22(6), 525–537.** [**https://doi.org/10.2165/00023210-200822060-00004**](https://doi.org/10.2165/00023210-200822060-00004) | Phillips, K. A., & Hollander, E. (2008). Treating body dysmorphic disorder with medication: Evidence, misconceptions, and a suggested approach. ***Body Image*,** ***5*(1), 13–27.** [**https://doi.org/10.1016/j.bodyim.2007.12.003**](https://doi.org/10.1016/j.bodyim.2007.12.003) |
| Fabricated | Phillips, K. A., et al. (2014). A meta-analysis of CBT for BDD. Psychotherapy, 51(2), 256–267. <https://doi.org/10.1037/a0036293> | Not found |
| Fabricated | Rosenfield, D., et al. (2018). CBT for BDD: A meta-analytic review. Journal of Obsessive-Compulsive and Related Disorders, 17, 132–141. <https://doi.org/10.1016/j.jocrd.2018.03.002> | Not found |
| Fabricated | Schneider, S. C., Turner, C. M., Mond, J., & Hudson, J. L. (2018). The role of family functioning in BDD symptoms in adolescents. European Child & Adolescent Psychiatry, 27(5), 615–624. <https://doi.org/10.1007/s00787-017-1089-3> | Not found |
| Fabricated | Summers, B. J., & Cougle, J. R. (2016). Acceptance and commitment therapy for BDD. Journal of Obsessive-Compulsive and Related Disorders, 11, 66–74. <https://doi.org/10.1016/j.jocrd.2016.09.001> | Not found |
| Non-fabricated but errors | Veale, D. (2004). **Cognitive–behavioral model of body dysmorphic disorder. Behaviour Research and Therapy, 42(5), 619–628. https://doi.org/10.1016/S0005-7967(03)00127-1** | Veale, D. (2004). **Advances in a cognitive behavioural model of body dysmorphic disorder.** ***Body Image*,** ***1*(1), 113–125. https://doi.org/10.1016/S1740-1445(03)00009-3** |
| Non-fabricated but errors | **Veale, D., et al.** (2014). **CBT vs anxiety management for BDD: RCT. British Journal of Psychiatry, 205(6), 499–507. https://doi.org/10.1192/bjp.bp.113.137058** | Veale, D., Anson, M., Miles, S., Pieta, M., Costa, A., & Ellison, N. (2014). **Efficacy of cognitive behaviour therapy versus anxiety management for body dysmorphic disorder: A randomised controlled trial.** ***Psychotherapy and Psychosomatics*,** ***83*(6), 341–353. https://doi.org/10.1159/000360740** |
| Fabricated | Veale, D., et al. (2016). The impact of BDD on quality of life. Journal of Affective Disorders, 205, 208–214. <https://doi.org/10.1016/j.jad.2016.08.020> | Not found |
| Non-fabricated but errors | **Wilhelm, S., Phillips, K. A., Greenberg, J. L., et al.** **(2014).** Cognitive-behavioral therapy for body dysmorphic disorder: A treatment manual. Guilford Press. | **Wilhelm, S., Phillips, K. A., & Steketee, G. (2013).** *Cognitive-behavioral therapy for body dysmorphic disorder: A treatment manual*. Guilford Press. |
| Non-fabricated but errors | Windheim, K., Veale, D., & Anson, M. (2011). **Mirror retraining for BDD: A pilot study. Behavioral and Cognitive Psychotherapy, 39(2), 271–276. https://doi.org/10.1017/S1352465810000722** | Windheim, K., Veale, D., & Anson, M. (2011). **Mirror gazing in body dysmorphic disorder and healthy controls: Effects of duration of gazing.** ***Behaviour Research and Therapy*,** *49* (9)**, 555–564. https://doi.org/10.1016/j.brat.2011.05.003** |
| Non-fabricated but errors | **Weingarden, H., et al**. (2017). **Shame mediates the relationship between BDD symptoms and suicidal ideation. Suicide and Life-Threatening Behavior, 47(5), 526–538. https://doi.org/10.1111/sltb.12307** | Weingarden, H., Renshaw, K. D., Davidson, E., & Wilhelm, S. (2017). **Relative relationships of general shame and body shame with body dysmorphic phenomenology and psychosocial outcomes.** ***Journal of Obsessive-Compulsive and Related Disorders*,** ***14*, 1–6. https://doi.org/10.1016/j.jocrd.2017.04.003** |
